# Mental health of healthcare workers in England during the first three years of the COVID-19 pandemic: The NHS CHECK study cohort

**DOI:** 10.1371/journal.pone.0350918

**Published:** 2026-06-26

**Authors:** Danielle Lamb, Hannah Scott, Ewan Carr, Sharon A.M. Stevelink, Rosalind Raine, Matthew Hotopf, Neil Greenberg, Pamela Almeida-Meza, Christopher Penfold, Sarah Ledden, Sarah Dorrington, Siobhan Hegarty, Ira Madan, Paul Moran, Richard Morriss, Dominic Murphy, Anne Marie Rafferty, Scott Weich, Simon Wessely

**Affiliations:** 1 University College London, London, United Kingdom; 2 King’s College London, London, United Kingdom; 3 University of Bristol, Bristol, United Kingdom; 4 University of Nottingham, Nottingham, United Kingdom; 5 University of Sheffield, Sheffield, United Kingdom; Fondazione Policlinico Universitario Agostino Gemelli IRCCS, Universita’ Cattolica del Sacro Cuore, ITALY

## Abstract

**Background:**

Maintaining healthcare workers’ (HCWs) mental health is vital to reduce staff absences and turnover, ultimately improving patient care. Most research focuses on clinical staff and single timepoints, overlooking non-clinical contributions.

**Aims:**

To examine mental health variations among all staff types over three years and identify those most at risk of poor mental health outcomes.

**Methods:**

Our prospective cohort study followed 22,092 HCWs from 17 English NHS Trusts. Online surveys assessed common mental disorders (CMDs), depression, anxiety, alcohol misuse, PTSD, moral injury, burnout, wellbeing, resilience, and post-traumatic growth at four timepoints from April 2020 to March 2023. Data were analysed cross-sectionally and weighted to represent Trust demographics.

**Results:**

Approximately 50% of participants reported probable CMDs at all timepoints. The most consistent predictor of poor mental health was having met the baseline cut-off for that outcome. No consistent differences emerged between clinical and non-clinical staff. Younger, female, lower-paid staff, those feeling unsupported by colleagues/managers, and exposed to morally injurious events were most at risk of poor mental health outcomes.

**Conclusions:**

All NHS staff types face persistent mental health struggles, with no significant improvement post-pandemic restrictions. Structural inequalities must be addressed long-term, alongside targeted, flexible support for staff in the short term.

## Background

Much has been written about the pressure the COVID-19 pandemic placed on healthcare systems, including the UK National Health Service (NHS). Health Care Workers (HCWs) are defined here as anyone working in a healthcare setting, including clinical and non-clinical roles. Non-clinical roles (e.g., porters, cleaners, receptionists) and non-patient-facing roles (e.g., HR, finance departments, administration) are as integral to the NHS, but largely excluded from research.

UK data show overall population mental health deteriorated during the pandemic, with significantly higher distress in April 2020 than previous years [1,2]. Systematic reviews of HCWs during COVID-19 report pooled prevalence estimates for anxiety symptoms of 30%, depression 31%, and Post-Traumatic Stress Disorder (PTSD) symptoms of 20% [3]. These align with levels seen in previous epidemics and pandemics [2,4]. However, there is conflicting evidence about whether this increase was more marked in HCWs. Using the UK Household Longitudinal Study (UKHLS; Understanding Society), a community study which was not selected by occupational group, Pierce et al. (2020) found no evidence to suggest that being a keyworker (including a HCW) was associated with an increase in mental distress.

General population studies suggest that being female and younger are risk factors for worse mental health [2,5]. There is also emerging evidence of associations between poor mental health and ‘potentially morally injurious events’ (PMIEs) [6], such as accidental or deliberate negligence, witnessing unethical behaviour and failing to intervene, or betrayal by trusted others (e.g., managers not providing appropriate support or resources), which may have been more likely during the pandemic. Our own and other research found that mental health outcomes are associated with peer/manager support, personal protective equipment (PPE) access, and job role and setting [7–9].

To date, most research has focused on clinical staff, limiting generalisability and overlooking non-clinical contributions. Many studies showing high HCW distress levels relied on single timepoint [7] and convenience samples, risking selection bias [10–12]. This study, NHS CHECK, addresses these limitations by inviting all HCW staff in participating Trusts to participate, enabling data weighting, and collecting data across nearly three years.

### Aims and hypotheses

The primary aim of this paper is to describe the NHS CHECK HCW cohort and their mental health outcomes across four timepoints, from April 2020 to March 2023 inclusive. Our main research question is: What is the prevalence of mental health outcomes in HCWs from April 2020 to March 2023.

The secondary aims of this paper are to investigate differences in mental health outcomes in NHS staff associated with: i) socio-demographic characteristics; ii) work setting; iii) job role; iv) occupational factors (e.g., access to PPE); v) organisational support available; and vi) exposure to potentially morally injurious events (PMIEs). Our secondary research question is: What factors are associated with worse mental health?.

Based on prior research, we hypothesise worse mental health outcomes during the pandemic among women, younger workers, those not in relationships, and Black and ethnic minority HCWs, as well as those with inadequate access to PPE, those lacking support from colleagues/managers, and those working in Intensive Care Units (ICU) and Accident & Emergency (A&E) settings [8]. We also hypothesised that those reporting greater exposure to PMIEs would report poorer mental health [13].

## Materials and methods

### Study design and participants

NHS CHECK is a longitudinal cohort study. The target population for NHS CHECK was all staff (clinical and non-clinical) working in 18 participating NHS Trusts. The full set of job roles included is listed in the Supplementary files. Data were collected using an online baseline survey between April 2020 and January 2021. Each participant was invited to complete follow-up surveys six, 12, and 32 months after their initial response. During the 12-month follow-up survey, a replenishment cohort was recruited from the same sites; these participants were missing baseline and six-month data. Collecting data at four timepoints for each participant balanced the need for longitudinal data against the pressure on staff [10]. Due to the large quantity of data available, this paper describes the cohort and mental health outcomes cross-sectionally at each of the four timepoints, while subsequent papers will analyse the data longitudinally, following individual trajectories across timepoints.

### Procedures

NHS Trusts were purposively selected to offer diversity in geographical location, urban and rural settings, and acute and mental health Trusts. Trusts were invited to participate via direct emails to senior leadership teams. Participating Trusts circulated emails explaining and promoting the study to all eligible staff via existing group email lists. A dedicated NHS CHECK recruitment email was sent by senior Trust management with a link to the study website (www.nhscheck.org), with variations of this email sent repeatedly during baseline recruitment. We also used existing staff support teams/leads, chief nursing officers, medical directors, occupational health departments, trade union representatives, and wellbeing hub users to promote the study. NHS CHECK was discussed during team briefings, included in Trust newsletters, news items on Trust intranet websites, closed social media groups, and advertised via screen savers on Trust computers. We fed back recruitment data to Trusts on a weekly basis, and provided incentives such as prizes (e.g., coffee machines) for Trusts with the highest proportions of recruitment. All participants who gave consent to be contacted again were entered into a prize draw to win one of 10 £50 and 10 £250 gift vouchers.

### Data collection and materials

The NHS CHECK Patient and Public Involvement (PPI) advisory group co-developed the questionnaire. The acceptability of the questions, materials and procedures was checked with a small group of frontline staff. Participants were shown an online information sheet, asked to complete an online consent form, and then completed an online questionnaire that took 5–10 minutes. At the end of the main survey, participants were given the option to complete a longer additional questionnaire (10–15 minutes).

The NHS CHECK baseline questionnaire was launched on the 24^th^ of April 2020, 5 weeks after the initial lockdown in the UK began (23/03/2020). Baseline recruitment closed on 15/01/2021, with the long roll-out period due to additional sites joining the study as it progressed. The six-month follow up survey was sent via email to participants six months after their baseline survey completion date, with a four-week window in which to complete the survey, and data collection closing on 15/08/2021. Similarly, the twelve-month follow up survey was sent to each participant 12 months after their baseline, with data collection closing on 21/02/2022. A 32-month survey was sent to all participants from 15/02/2023, with data collection closing on 02/05/2023.

#### Measures.

At baseline and the six- and 12-months follow-ups, in addition to the main questionnaire, participants were invited to complete an additional longer survey. Splitting the survey offered participants the option to reduce the time taken to contribute, while still collecting some key data. As a result, sample sizes for measures included in the ‘long’ survey are smaller than the overall sample size. At 32-months all participants received the full set of survey questions. While the majority of the survey at each time used the same measures, to account for changing research and policy priorities the surveys were not identical. See the Supplementary files for information on which measures were used in each survey.

Surveys collected information on participants’ socio-demographic characteristics, their occupational role and context, COVID-19 experiences, and the validated Moral Injury Events Scale (MIES) was used to collect data on exposure to potentially morally injurious events (PMIEs) [14], with endorsement of one or more item indicating exposure (i.e., selecting moderately/strongly agree).

The primary outcome was prevalence of probable common mental disorders (CMDs), ascertained using the 12-item General Health Questionnaire (GHQ-12). The GHQ scoring method was used (where each item is scored 0-0-1-1, resulting in a total score of 0–12 for the scale), with a cut-off score of 4 or more indicating ‘caseness’ of a CMD (indicating increased probability of experiencing a recognised mental disorder) [15].

Secondary outcomes included:

1. Anxiety, assessed using the Generalised Anxiety Disorder scale (GAD-7) with higher scores indicating higher levels of anxiety, and a cut-off of ≥10 indicating caseness [16]
2. Depression, assessed using the Patient Health Questionnaire (PHQ-9) with higher scores indicating higher levels of depression, and a cut-off of ≥10 indicating caseness [17].
3. Problem drinking, assessed using the Alcohol Use Disorder Identification Test (AUDIT-C), with higher scores indicating more problematic drinking, and a cut-off of ≥8 indicating caseness [18].
4. Post-Traumatic Stress Disorder (PTSD), assessed using the Post-Traumatic Stress Disorder checklist (PCL–6), with higher scores indicating higher levels of PTSD, and a cut-off of ≥14 indicating probable caseness [19].
5. Burnout, assessed using the Burnout Assessment Tool (BAT-12), with higher scores indicating higher levels of burnout, and a cut-off of 2.96 indicating probable caseness [20].
6. Wellbeing, assessed using the Warwick Edinburgh Mental Wellbeing Scale (WEMWBS), with higher scores indicating better mental health, and scores of ≥43 indicating good mental health [21].
7. Resilience, assessed using the Brief Resilience Scale (BRS), with higher scores indicating higher levels of resilience [22].
8. Growth after trauma, assessed using the Post-Traumatic Growth Inventory Short Form (PGTI-SF), with higher scores indicating higher levels of post-trauma growth [23].

There are no accepted cut-off scores for the BRS or PTGI, so, for the purposes of consistent analysis, for this paper we generated tertiles for each measure and used the cut point between the top and middle tertiles in each case to indicate presence of high resilience or growth respectively. Using this method, for the BRS a score of ≥24 indicates higher levels of resilience, and for the PTGI a score of ≥17 indicates higher levels of growth.

#### Survey period.

To account for time-varying pressures on NHS Trusts and staff, we included a variable measuring survey period. We hypothesised that reporting of mental ill-health symptoms would be greater during periods of higher pressure and that this could impact on outcomes at later timepoints. In line with previous research [24], we identified pressure as: High early in the pandemic (T1: April-June 2020), when little was known about COVID-19 and swift changes were needed in healthcare settings and in general life; Lower in the summer/early autumn of 2020 (T2: July-October 2020), when the initial wave of infections lessened and lockdown restrictions eased; before increasing again over the winter months (T3: November 2020-February 2021). These time periods correspond to periods with higher and lower COVID-19 death rates: T1 COVID-19 deaths = 53,389; T2 COVID-19 deaths = 7,157; T3 COVID-19 deaths = 82,083 [25]. This enabled us to explore outcomes and associated factors, taking into account the effects of differing levels of pressure on the NHS when participants completed their baseline and some of the follow-up surveys.

### Statistical analysis

#### Data cleaning.

Using email address as a unique identifier, where participants erroneously completed the survey more than once (at any timepoint), we retained their first response only, with any missing demographic data available in dropped responses added to the retained response.

As specified in the protocol [26], we excluded any Trust where the response rate was below five percent.

#### Weighting.

We derived two sets of weights. First, we generated baseline response weights using aggregate demographic information provided by Trust Human Resources (HR) departments as of April 2020. We derived weights for the full baseline cohort using a raking algorithm (citation) based on age, sex, ethnicity, job role For weighting purposes only, missing demographic data were imputed using k-Nearest Neighbour (kNN) imputation (k = 5; [27]). At baseline, missingness was ≥ 5% for age, sex, ethnicity, and job role. Individual person level strata were weighted by Trust size as well as composition of the four main proposed prognostic factors of ethnicity, categorical age, role and sex. Post strata and post weight specifications were included but no finite population correction was made.

Second, we applied inverse probability weighting to account for non-response at each follow-up timepoint. A binary logistic regression model was used to model the probability of non-response based on age, sex, ethnicity, and job role at each timepoint.

For each timepoint, we multiplied these two weights to create a single combined weight that was applied to all analyses.

#### Descriptive analyses.

We explored the data in several stages. First, we described the characteristics of those completing the surveys at each timepoint. We then summarised the weighted prevalence and mean scores of the primary (GHQ-12) and secondary outcomes (GAD-7, PHQ-9, AUDIT-C, PCL-6, BAT-12, WEMWBS, BRS, PTGI) cross-sectionally by timepoint. Finally, we checked for collinearity between the variables.

#### Regression analyses.

We used multilevel models to account for clustering by Trust. Our primary interest was in exploring factors associated with meeting cut-off scores on our outcome variables, so we used multivariable logistic regression models. The factors included in the models were identified *a priori*, based on existing evidence and research team discussions. The baseline factors included in the models were agreed upon by the research team based on existing evidence and expert opinion: age category; sex; ethnicity; relationship status; job role; pay grade; job setting; had good access to PPE; felt supported by colleagues; felt supported by managers; experienced moral injury; baseline measure of the outcome variable (in models for 6, 12, and 32 months); the survey period during which participants completed their baseline survey. We modelled outcomes adjusting for all other variables in the model, and include unadjusted models in Table S3 in S1 File.

This paper presents descriptive data at multiple time points, and we provide longitudinal trajectory modelling in a separate paper [accepted for publication in Psychological Medicine – reference to be provided ASAP].

All analyses were conducted using Stata version 18.0.

### Ethical approval

Ethical approval for the study was granted by the Health Research Authority (reference: 20/HRA/2107, IRAS: 282686) and local Trust Research and Development approval. The study was approved as having Urgent Public Health Status in August 2020 and was conducted in accordance with the Declaration of Helsinki. Clinical trial number: not applicable. Written consent was obtained digitally from participants, via ticking boxes next to each consent statement in an online form at the start of the survey.

## Results

The baseline survey included 17 NHS Trusts with a total population size of 139,037. From these trusts, 26,088 people responded to the initial baseline survey, representing an overall response rate of 16% (ranging from 5% to 55% across Trusts). After excluding empty responses (i.e., where someone opened the questionnaire but did not provide any data; n = 167) and duplicate responses (n = 1,304), the sample size was 24,617. We excluded respondents from one Trust where the response rate was below five percent (n = 514) and those who did not report a Trust (n = 2,011; necessary in order to weight the data), giving a final baseline sample size of 22,092. Table 1 presents the sample size at each time period, and the numbers completing the short survey only, compared to the numbers completing both the short and long surveys.

**Table 1:** Number of participants completing short and long surveys at each time period.

	Baseline (0 months)	6 months	12 months^a^	32 months
**Short survey only**	9,799	5,428	5,531	N/A
**Short and long surveys**	12,293	5,258	6,467	6,991
**Total**	22,092	10,514	11,998	6,991

^a^At 12-months, the sample size was 10,429, plus an additional 1,569 new participants who were recruited at that timepoint as the replenishment cohort (but for whom baseline and six-month data are missing).

We assessed differences between responders and non-responders by key demographics (age, sex, ethnicity, job role) at six, 12, and 32 months, using T tests for continuous variables and Chi2 for categorical variables. At all three time points, responders (vs non-responders) were likely to be older by around 3–5 years (p < 0.001). At six months, responders were more likely to be women (46% vs 44%, p = 0.022), though there were no statistically significant differences in responders by sex at 12 or 32 months. At all three time points, responders were more likely to be White, and in the non-clinical staff group, and though statistically significant, differences were small. Full details can be found in the Supplementary files.

### Descriptive analyses

#### Cohort demographics.

The demographic composition of the sample can be seen in Table 2, which shows the total number of respondents in each category, with total unweighted and weighted proportions given, for the four time periods.

**Table 2:** Descriptive characteristics of the cohort for all time periods, with weighted and unweighted proportions.

Characteristic	Baseline (n = 22,092)			6 months (n = 10,514)			12 months (n = 11,998)			32 months (n = 6,991)		
	n	Unweighted %	Weighted %	n	Unweighted %	Weighted %	n	Unweighted %	Weighted %	n	Unweighted %	Weighted %
Age (years) mean (SD)		M 43 (SD 12)	M40 (SD 12)		M 45 (SD 12)	M42 (SD 12)		M 45 (SD 12)	M42 (SD 12)		M 46 (SD 11)	M 43 (SD 12)
≤ 30	4,230	19	21	1,610	15	19	1,823	15	20	804	12	17
31-40	4,848	22	25	2,134	20	24	2,253	19	22	1,311	19	23
41-50	5,522	25	22	2,759	26	21	3,177	27	21	1,984	28	22
51-60	5,147	23	20	2,835	27	20	3,365	28	20	2,058	29	19
≥ 61	1,303	6	7	744	7	6	885	7	6	543	8	6
Missing data	1,042	5	5	432	4	10	495	4	11	291	4	13
Sex												
Female	17,726	80	74	8,519	81	74	9,694	81	73	5,572	80	73
Male	4,116	19	25	1,934	18	25	2,213	18	25	1,352	19	24
Missing data	250	1	1	61	1	1	91	1	2	67	1	3
Relationship status												
Single	5,706	26	27	2,618	25	26	3,114	26	28	1,783	26	27
In a relationship	16,162	73	72	7,841	75	73	8,804	73	71	5,148	74	71
Missing data	224	1	1	55	<1	1	80	1	1	60	<1	2
Ethnicity												
White	18,738	85	75	9,270	88	77	10,592	88	77	6,191	89	75
Black/African/Caribbean												
/Black British	971	4	9	332	3	7	406	3	7	216	3	6
Asian/Asian British	1,458	7	12	562	5	11	573	5	11	330	5	12
Mixed/Multiple racial and ethnic groups	537	2	1	235	2	1	277	2	1	152	2	1
Other racial and ethnic minority groups	205	1	2	80	1	3	88	1	3	57	<1	3
Missing data	183	1	1	35	<1	<1	62	1	1	45	<1	2
Main role												
Doctor	1,613	7	10	741	7	11	776	7	10	416	6	9
Nurse	5,652	26	29	2,580	25	28	2,983	25	28	1,736	25	29
Other clinical	6,654	30	31	3,027	29	31	3,515	29	31	1,975	28	30
Non-clinical	8,012	36	29	4,139	39	30	4,643	39	30	2,808	40	29
Missing data	161	<1	1	27	<1	<1	81	<1	1	56	1	2
Job setting												
A&E	331	2	3	151	1	3	164	1	3	83	1	2
ICU/Anaesthetics	790	4	5	421	4	6	391	3	5	231	3	6
Other hospital	12,662	57	66	6,195	59	66	6,739	56	63	3,946	56	64
Community	6,543	30	19	2,978	28	19	3,415	29	19	2,025	29	19
Non-patient-facing	1,034	5	4	578	6	5	517	4	4	327	5	4
Missing data	732	3	3	191	2	1	772	6	6	379	5	6
PPE access often/always												
Yes	16,755	78	78	7,957	76	78	7,945	66	68	469	69	70
No	1,675	9	9	783	7	9	738	6	7	4,841	7	8
Missing data	3,662	14	14	1,774	17	13	3,315	28	25	1,681	24	22
Felt supported by colleagues												
Yes	19,630	89	88	9,587	91	91	9,382	78	78	482	82	81
No	1,654	8	8	786	8	8	784	7	7	5,752	7	8
Missing data	808	3	4	141	1	1	1,832	15	15	757	11	11
Felt supported by manager												
Yes	17,835	79	78	8,728	83	81	8,521	71	70	988	14	17
No	3,425	18	18	1,634	16	17	1,635	14	15	5,240	75	72
Missing data	832	3	4	152	1	1	1,842	15	15	763	11	11
Pay grade^a^												
AfC 5 or below	6,975	28	28	3,397	32	27	3,786	32	28	2,263	32	28
AfC 6 or above (inc. medical pay scales)	11,880	55	54	5,848	56	57	6,276	52	54	3,740	53	55
Missing data	3,237	17	18	1,269	12	16	1,936	16	18	2,968	14	17
Experienced Moral Injury^b^												
Yes	2,847	14	14	1,676	16	18	1,633	14	15	3,019	14	16
No	8,569	37	37	4,986	47	45	4,817	40	38	1,004	43	40
Missing data^c^	10,586	49	49	3,852	37	37	5,548	46	47	2,968	43	44
Survey period^d^												
Time 1 (Apr-June 20)	5,423	25	25	3,074	29	29	2,534	21	24	1,595	23	25
Time 2 (July-Oct 20)	11,309	56	56	5,035	48	52	5,749	48	50	3,303	47	49
Time 3 (Nov 20 – Feb 21)	5,360	20	19	2,405	23	19	2,146	18	14	1,509	22	18
Missing data^e^	–	–	–	–	–	–	1,569	13	12	584	8	8

^a^ Pay scale is dichotomised at approximately the median national average wage in the UK, £30,472 [28], using the Agenda for Change (AfC) pay scales [29] and medical pay scales [30].

^b^ Data was collected on moral injury at each of the four timepoints. All other variables were collected at baseline, and this data presents the numbers and proportions of each characteristic who provided data at subsequent timepoints.

^c^ Missing data for Moral Injury is high due to this measure appearing in the long questionnaire, which was completed by around half of participants.^d^ Survey period refers to the time period during which participants completed their baseline survey.

^e^ The replenishment cohort recruited at 12 months (n = 1,569) did not complete baseline or 6 month surveys, hence there is data for all participants on ‘Survey period’ at baseline and 6 months, but up to 1,569 participants with missing data at 12 and 32 months.

Abbreviations: A&E = Accident and Emergency; ICU = Intensive Care Unit; PPE = Personal Protective Equipment; M = Median; SD = Standard Deviation.

On checking for collinearity between the variables, the mean VIF was 1.16, and no VIF was found above 1.5, indicating that collinearity is not a concern for these variables.

#### Outcome prevalence.

At baseline, over half of participants reported symptoms of common mental disorders (CMDs) (52%, 95%CI 51%, 53%), and this was very similar at six months (51%, 95%CI 49%, 52%), 12 months (47%, 95%CI 46, 49), and 32-months (50%, 95%CI 48%, 52%). The proportion of those reporting symptoms of anxiety and depression remained stable over the four time periods, at between 21–29%, and the proportion reporting good wellbeing also showed little change, at around 50% at each time period. Proportions reporting alcohol misuse and PTSD showed a spike from baseline to six months (around 1/5 and 1/3 reporting symptoms respectively) before dropping again at 12 and 32 months, although notably PTSD remained at nearly 1/3 at 32 months. Proportions reporting burnout rose over the first three time periods, from 16% (95%CI 14%, 17%) at baseline to 26% (95%CI 24%, 27%) at 12 months, dropping to 20% (95%CI 19%, 22%) at 32 months. Levels of resilience remained at around 1/3 reporting resilience at the first three timepoints. PTGI was collected at 6,12, and 32 months, and remained at just over 1/3 reporting growth at each timepoint.

**Fig 1:**
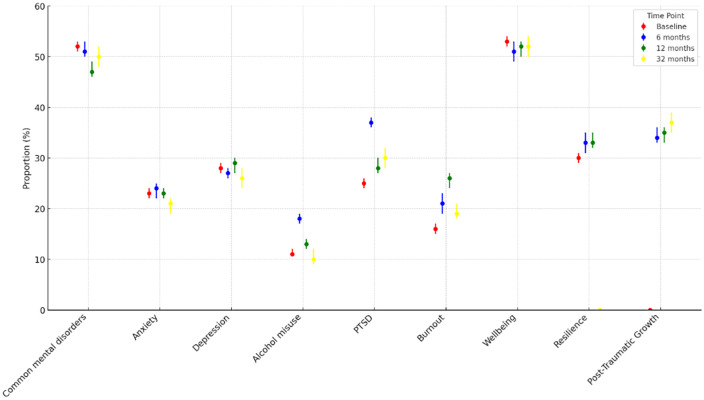
Dot plot of outcome variables across timepoints with 95% confidence interval whiskers.

Fig 1 shows proportions and confidence intervals for each outcome measure (this data is available in Table S2 in S1 File).

### Regression analyses

#### Common mental disorders (primary outcome).

A multilevel multivariable logistic regression found that at baseline, six and 32 months, compared to those aged 30 or younger, those over 40 had statistically significantly lower odds of reporting symptoms of CMDs, but this was not the case at 12 months, where 31–40 year olds had higher odds than those ≤30. At baseline, six months, and 32 months, compared to women, men had lower odds of reporting symptoms of CMDs. Compared to White participants, at baseline Asian participants had lower odds of reporting symptoms of CMDs, and at 32 months those in a relationship had lower odds of reporting symptoms than those who were single. At baseline and 12 months, compared to non-clinical staff, those in other clinical roles had higher odds of reporting symptoms, while at six months doctors had lower odds of reporting symptoms than non-clinical staff. At six months, those working in ICU/Critical Care had higher odds of reporting symptoms. At baseline and 32 months, those who often or always had access to adequate PPE had lower odds of reporting symptoms than those who never, rarely, or sometimes had access. At baseline, those who felt supported by colleagues and by managers had lower odds of reporting symptoms than those who did not feel supported, and this was true for colleagues at 12 months too. At all timepoints, those who had experienced moral injury had higher odds of reporting symptoms than those who had not. Those who completed their baseline survey later, during a high pressure period (Time 3, Nov 20-Feb 21) had higher odds of reporting symptoms at baseline and 12 months, compared to those who completed their baseline survey during a lower pressure period (Time 2, Jul-Oct 2020). Full results are available in Table 3.

**Table 3:** Weighted, multilevel, multivariable binary logistic regression models of factors associated with meeting GHQ-12 cut-off at each timepoint.

	Baseline (n = 8,050) AOR (95%CI) p-value	6 months (n = 4,142) AOR (95%CI) p-value	12 months (n = 4,234) AOR (95%CI) p-value	32 months (n = 2,658) AOR (95%CI) p-value
**Age (years) (ref: ≤ 30)**				
31-40	0.87 [0.71 1.07] 0.18	1.10 [0.89 1.35] 0.36	**1.71 [1.11, 2.63] 0.02**	1.22 [0.99, 1.51] 0.06
41-50	**0.75 [0.57 0.97] 0.03**	**0.75 [0.62 0.92] 0.01**	1.21 [0.85, 1.74] 0.27	0.93 [0.79, 1.10] 0.37
51-60	**0.68 [0.53 0.88] 0.01**	**0.77 [0.61 0.98] 0.03**	1.42 [0.92, 2.18] 0.10	**0.85 [0.74, 0.97] 0.02**
≥ 61	**0.51 [0.41 0.64] <0.01**	**0.58 [0.40 0.83] 0.01**	1.36 [0.71, 2.60] 0.33	**0.70 [0.49, 1.00] 0.05**
**Sex (ref: Female)**				
Male	**0.75 [0.61 0.92] 0.01**	**0.86 [0.74 1.00] 0.05**	0.91 [0.70, 1.19] 0.47	**0.78 [0.62, 0.98] 0.03**
**Ethnicity (ref: White)**				
Black	0.79 [0.54 1.15] 0.21	0.71 [0.36 1.40] 0.30	0.79 [0.46, 1.34] 0.36	1.02 [0.61, 1.70] 0.94
Asian	**0.64 [0.45 0.92] 0.02**	0.73 [0.46 1.18] 0.19	1.06 [0.70, 1.60] 0.76	1.16 [0.78, 1.73] 0.44
Mixed	1.43 [0.84 2.44] 0.17	1.33 [0.65 2.74] 0.41	2.23 [0.81, 6.15] 0.11	2.42 [0.93, 6.33] 0.07
Other	1.04 [0.33 3.28] 0.94	1.38 [0.82 2.31] 0.21	1.91 [0.96, 3.81] 0.07	1.57 [0.51, 4.80] 0.41
**Relationship (ref: Single)**				
In a relationship	0.97 [0.83 1.14] 0.70	0.99 [0.72 1.36] 0.94	0.84 [0.62, 1.13] 0.24	**0.67 [0.56, 0.80] 0.00**
**Role (ref: Non-clinical)**				
Doctor	1.22 [0.36 4.16] 0.73	**0.35 [0.17 0.72] 0.01**	0.85 [0.30, 2.40] 0.75	0.17 [0.02, 1.48] 0.10
Nurse	1.07 [0.85 1.34] 0.53	0.98 [0.76 1.26] 0.85	1.18 [0.82, 1.69] 0.35	1.14 [0.89, 1.45] 0.29
Other clinical	**1.14 [1.02 1.28] 0.03**	1.02 [0.76 1.36] 0.88	**1.30 [1.03, 1.64] 0.03**	0.94 [0.64, 1.40] 0.75
**Pay grade (ref: ≤ AfC grade 5)**				
≥ AfC grade 6	1.17 [0.98 1.40] 0.08	0.89 [0.60 1.32] 0.53	0.83 [0.55, 1.24] 0.34	**0.96 [0.67, 1.38] 0.82**
**Job setting (ref: Other hospital)**				
A&E	0.74 [0.43 1.29] 0.27	2.26 [0.82 6.20] 0.11	1.10 [0.43, 2.82] 0.83	2.29 [0.66, 7.98] 0.18
ICU/Critical Care	1.38 [0.93 2.04] 0.10	**1.95 [1.32 2.88] <0.01**	1.30 [0.86, 1.97] 0.20	1.03 [0.46, 2.30] 0.93
Community	1.23 [0.98 1.53] 0.07	1.20 [0.95 1.51] 0.13	0.90 [0.76, 1.08] 0.23	1.07 [0.87, 1.30] 0.51
Non-patient facing	0.97 [0.74 1.27] 0.80	0.60 [0.31 1.18] 0.13	0.68 [0.37, 1.23] 0.19	0.89 [0.38, 2.10] 0.78
**PPE access (ref: never/sometimes)**				
Often/always	**0.71 [0.58 0.87] <0.01**	1.05 [0.77 1.43] 0.75	0.89 [0.55, 1.43] 0.60	**0.60 [0.39, 0.90] 0.02**
**Colleague support (ref: not at all/a little)**				
Moderately/ Extremely	**0.51 [0.36 0.73] <0.01**	0.69 [0.41 1.16] 0.15	**0.59 [0.41, 0.85] 0.01**	1.12 [0.67, 1.88] 0.64
**Manager support (ref: not at all/a little)**				
Moderately/ Extremely	**0.49 [0.42 0.58] <0.01**	1.02 [0.80 1.28] 0.89	0.84 [0.59, 1.21] 0.33	1.04 [0.71, 1.50] 0.84
**Moral injury (ref: below cut-off)**				
Met cut-off (experiencing ≥1 PMIE)	**1.94 [1.60 2.35] <0.01**	**1.26 [1.05 1.52] 0.02**	**1.40 [1.10, 1.79] 0.01**	**1.47 [1.22, 1.77] 0.00**
**Survey period (ref: Time 2 July-Oct 20)**				
Time 1 (Apr-June 20)	1.37 [0.97, 1.95] 0.07	1.13 [0.82, 1.56] 0.42	0.79 [0.55, 1.13] 0.17	0.76 [0.47, 1.21] 0.23
Time 3 (Nov20 – Feb 21)	**1.37 [1.17, 1.60] <0.01**	0.85 [0.67, 1.98] 0.16	**1.42 [1.07, 1.99] 0.02**	0.83 [0.58, 2.47] 0.26
**Baseline GHQ**				
Met cut-off at baseline	**–**	**3.81 [2.69 5.38] <0.01**	**3.43 [2.44, 4.81] 0.00**	**2.77 [2.09, 3.66] 0.00**

Odds ratios shown are adjusted for all other covariates in each model.

#### Secondary outcomes.

The full results of the secondary outcome models are available in Supplementary files (Tables S4-S11 in S1 File). A summary of the results is available in Table 4 below.

**Table 4:** Summary of primary and secondary outcome results – those with higher odds of meeting cut-off (unless otherwise specified).

	Baseline	6 months	12 months	32 months
Common Mental Disorders (GHQ)	• Younger • Female • Asian < White • Other clinical • Insufficient PPE • Not supported by colleagues & manager • Moral injury • Time 3	• Younger • Female • Doctor < Non-clinical • ICU/Critical care setting • Moral injury • Met baseline cut-off	• Younger • Other clinical • Not supported by colleagues • Moral injury • Time 3 • Met baseline cut-off	• Younger • Female • Single • Lower paid • Insufficient PPE • Moral injury • Met baseline cut-off
Anxiety (GAD-7)	• Younger • Female • Asian < White • Single • Lower paid • Non-patient-facing setting • Not supported by manager • Moral injury • Time 3	• Younger • Asian < White • Mixed ethnicity > White • Not supported by colleagues • Moral injury • Time 1 • Met baseline cut-off	• Younger • A&E setting • Not supported by colleagues • Moral injury • Met baseline cut-off	• Younger • Single • Doctor < Non-Clinical • Insufficient PPE • Met baseline cut-off
Depression (PHQ-9)	• Younger • Single • Lower paid • No PPE • Not supported by colleagues & manager • Moral injury • Time 1 & 3	• Younger • Black < White • A&E setting • Not supported by colleagues • Moral injury • Time 1 • Met baseline cut-off	• Younger • Nurse • Lower paid • A&E setting • Community setting < Other hospital setting • Insufficient PPE • Moral injury • Met baseline cut-off	• Younger • Single • ICU/Critical care setting • Insufficient PPE • Moral injury • Met baseline cut-off
Alcohol Misuse (AUDIT)	• Older • Male • White	• Asian < White • Non-clinical • Higher paid • Time 3 • Met baseline cut-off	• Male • A&E setting < • Nurse < Non-clinical • ICU/Critical care setting • Met baseline cut-off	• Black < White • Doctor < Non-clinical • Met baseline cut-off
PTSD (PCL-6)	• Younger • Female • Asian • Lower paid • ICU setting • Insufficient PPE • Moral injury • Time 3	• Younger • Female • Moral injury • Time 3 < • Met baseline cut-off	• Nurse • A&E & ICU/Critical care settings • No PPE • Time 3 • Met baseline cut-off	• Younger • Asian < White • Single • Met baseline cut-off
Burnout (BAT)	• Younger • Not supported by colleagues & manager • Moral injury • Time 3	• Younger • ICU/Critical care setting • Moral injury • Time 1 & 3 • Met baseline cut-off	• Younger • Other Clinical • ICU & Community settings • Not supported by colleagues & manager • Met baseline cut-off	• Younger • Black & Asian < White • Single • Higher paid • A&E setting < Other hospital setting • Moral injury • Met baseline cut-off
Wellbeing (WEMWBS)	• Younger • White • Single • Lower paid • Insufficient PPE • Not supported by colleagues & manager • Moral injury	• Younger • Moral injury • Met baseline cut-off	• Community settings < Other hospital settings • Moral injury • Met baseline cut-off	• Younger • Mixed ethnicity • Black < White • Single • Non-patient-facing setting • Insufficient PPE • Moral injury • Did not meet baseline cut-off
Resilience (BRS)	• Younger • Black < White • Single • Lower paid • Moral injury • Time 3	• Younger	• Nurse < Non-clinical • Higher paid • Met baseline cut-off	Not collected at 32 months
Post- Traumatic Growth (PTGI)	Not collected at baseline	• Male • White < Asian & Other ethnicity • Other hospital < Community setting • Not supported by manager • Moral injury	• Male • White < Black & Asian • Single • Other hospital < Community setting • Met baseline cut-off	• White < Other ethnicity • Non-clinical < Doctor & Nurse • Higher paid • Met baseline cut-off

The strongest and most consistent finding was that having met cut-off on a measure at baseline was associated with meeting cut-off on that same measure at the current time. This was the case for nearly all timepoints and all measures, except that those reporting more resilience at baseline did not have higher odds of reporting more resilience at 6 months.

Several factors were associated with nearly every measure, but not at every timepoint. Being younger was associated with worse outcomes across every measure at some timepoint, particularly at baseline and 6 months. Older age was associated (at baseline) with alcohol misuse. Moral injury was consistently associated with worse outcomes across all measures except alcohol misuse. Feeling unsupported by colleagues/managers was linked to worse outcomes for most measures, excluding alcohol misuse, resilience, and post-traumatic growth. Being single was also associated with worse outcomes across most measures.

Black, Asian, Mixed and Other ethnicity participants generally had better outcomes than White participants, across multiple measures and timepoints. Exceptions included worse alcohol outcomes for White participants at all timepoints except 12 months.

Female participants had worse outcomes across most measures and across timepoints, except for alcohol misuse (more in males), and greater post-traumatic growth. Notably, females did not report worse depression than males at any time.

Findings were less consistent for other factors. Job setting showed mixed results, with worse outcomes for A&E and ICU/Critical care on some measures at various timepoints, and those working in other hospital settings, community, and non-patient-facing settings having worse outcomes on other measures/timepoints.

There was some evidence that inadequate PPE was associated with worse outcome. Similarly, nurses, other clinical staff, and non-clinical staff reported reported worse outcomes than doctors on some measures at some timepoints.

Those who completed their baseline survey at a higher pressure timepoint (Time 1 and 3) tended to have worse outcomes than those at Time 2.

Lower pay grade was associated with worse outcomes on some measures at different timepoints. However, higher paid was associated with higher alcohol misuse at 6 months, lower resilience at 12 months, and higher burnout at 32 months.

In the summary table below, the characteristics listed are those with higher odds of having poor outcomes, unless otherwise specified.

## Discussion

### Summary of findings

We followed a national cohort of 22,501 HCW participants, weighted to represent local Trust demographics, and found that prevalence of probable common mental disorders remained relatively stable throughout the pandemic, with around 50% of participants reporting symptoms at any given time.

The most consistent predictor across outcomes was meeting the cut-off for that outcome at baseline, which was the case for every measure at every timepoint, except resilience at six months. Younger age, lack of support from colleagues or managers, greater exposure to morally injurious events, and completing the baseline survey at a higher-pressure time were associated with higher odds of mental disorder symptoms and lower odds of wellbeing, resilience, and post-traumatic growth. There was some evidence that being female, White, or single was associated with poorer outcomes. Findings regarding job role, setting, and pay grade were mixed.

### Findings in relation to previous research

The UK Household Longitudinal Study (UKHLS) found that probable CMD prevalence in the general population was significantly higher during the early pandemic months (29.5% in April 2020 vs. 20.8% in April 2019, 28.0% in May 2020, and 26.9% in June 2020), with distress most pronounced for those aged 18−34 and female [5]. Analyses found no statistically significant increases in GHQ-12 score in keyworkers (e.g., HCWs, teachers) early in the pandemic [2], and no significant differences in anxiety and depression growth trajectories between key worker and non-keyworkers [31].

We found a higher prevalence of psychological distress in our HCW sample (baseline GHQ: 52%, 95%CI 51, 53) compared to previous general population studies (<30% [5]). Occupational surveys may report higher distress prevalence than general population surveys, likely due to reporting or contextual bias [32].

Our findings align with previous research highlighting workplace support as crucial for wellbeing [33] and the impact of morally injurious events [6]. Younger age predicted poorer outcomes, consistent with general population surveys [2], though at 12 months, those aged 31−40 had poorer outcomes, possibly due to caregiving responsibilities. Female sex was associated with poorer outcomes, aligning with UK and international research [9,34–39], particularly relevant given that 76% of the NHS workforce are women [40]. Gender inequalities in caregiving responsibilities and workplace structures (e.g., PPE designed for average male sizes) [41] may contribute. However, measures like the GHQ-12 may not capture distress in men [2]. Alcohol use measures may better reflect distress in men, and our data showed men were more likely than women to report alcohol misuse at baseline and 12 months.

Our hypothesis that ICU staff would be most affected was partially supported: ICU and A&E staff showed higher odds of worse mental health outcomes. Other studies have reported high PTSD levels in ICU staff [7], possibly predating the pandemic. However, we lack data on index trauma events [42]. Staff in other settings had lower odds of reporting positive mental health outcomes (e.g., wellbeing).

Few significant associations were found between ethnicity and wellbeing, possibly due to small numbers and lower engagement from those most likely to report negative outcomes. This is surprising given evidence of discrimination and harassment affecting job satisfaction and sickness absence [43]. The clearest ethnic association was with alcohol use: Black, Asian, and mixed racial or ethnic groups were less likely to meet the cut-off for alcohol misuse across all timepoints compared to White participants, consistent with other research [44].

The most consistent predictor of an outcome was meeting its cut-off at baseline, reinforcing the long-established finding that current clinical state is the best predictor of future clinical state [45]. This highlights the importance of prevention and early intervention.

### Strengths and limitations

Our study’s key strengths are its size and comprehensiveness, with data from over 22,000 NHS staff across 17 acute and mental health NHS Trusts in diverse urban and rural areas. Data were weighted to represent each Trust’s demographics and account for non-response. The study’s longitudinal design offers insight into HCW mental health and well-being over three years. Analyses accounted for data clustering via multi-level models. These strengths provide, to our knowledge, the best quality data available regarding HCW mental health and wellbeing.

Limitations include data collection timing. Each wave lasted ~10 months to maximize response rates, meaning data were collected at different pandemic stages. We addressed this by presenting cross-sectional data and including survey period as a variable in regression models.

We lack pre-pandemic data, preventing assessment of prior ill health or the already rising CMD rates in younger age groups. Baseline data were collected from April 2020, when population surveys already showed poorer mental health. Additionally, we lack staff attrition data, making it unclear how many participants left the NHS or why. We do, however, have data on attrition within the study itself, and provide analyses in the Supplementary files. The differences between responders and non-responders at the three follow up time points were all small, though some were statistically significant.

Although our 16% baseline response rate exceeded similar studies, it was suboptimal, and may have either overrepresented or underrepresented distressed participants. On balance, given that those most distressed are likely to exit the workforce either temporarily or permanently, it seems more likely that results underrepresent than overrepresent prevalence. However, we weighted the data for representation and non-response at later timepoints. Recruitment via emails may have excluded agency staff and those without regular computer access.

To reduce participant burden, we split the survey at baseline, six, and 12 months into a mandatory short and an optional long version, completed by ~50% of participants. This may introduce selection bias but helped minimise burden. Finally, self-report measures indicate symptom prevalence rather than diagnosed mental disorders [46].

### Implications for future research, policy and practice

All types of staff experienced similar distress levels, suggesting that early mental health support should extend beyond clinical and frontline roles. Trusts should consider specific needs of younger staff and the differing support needs by sex. Meeting baseline measure cut-offs predicted poorer outcomes, underscoring the importance of prevention, detection, and targeted early intervention.

Since pandemic measures ended, NHS Trusts have faced staff mental health budget cuts, leaving occupational health as the primary resource. Our findings suggest mental health did not improve as restrictions lifted; in some cases (PTSD and burnout) it worsened. While some staff found motivation and teamwork during the pandemic, many remain distressed by ongoing challenges [47,48].

Our findings on workplace support indicate that enabling NHS supervisors to support teams and foster good team dynamics is key to staff mental health [49]. Leaders also need support, and a cascading model help. Future pandemic planning should prioritise early PPE policies to mitigate mental distress.

## Conclusions

This study provides the most comprehensive evidence available on HCW mental health and wellbeing over a 32-month period, with strengths including its defined sampling frame, large sample size, and weighting for representativeness and attrition. Our findings highlight that all NHS staff—not just clinical roles—are struggling, a previously underexplored issue. However, specific trauma-prone settings like ICU and A&E may pose higher risks. Younger, female, lower-paid staff, those who feel unsupported, and those exposed to morally injurious events are most at risk of negative mental health outcomes. Addressing structural inequalities and providing targeted short-term support are crucial moving forward.

## Supporting information

S1 File Please see ‘NHS Check full cohort Supplementary files FINAL.docx’ for supporting information. (DOCX)

## Abbreviations

A&E: Accident and Emergency
AOR: Adjusted Odds Ratio
AUDIT: Alcohol Use Disorder Identification Test
BAT: Burnout Assessment Tool
BRS: Brief Resilience Scale
CI: Confidence Interval
CMD: Common Mental Disorder;
GAD: Generalised Anxiety Disorder
GHQ: General Health Questionnaire
HCW: Healthcare Worker
HR: Human Resources
ICU: Intensive Care Unit
NHS: National Health Service
PCL: Post-Traumatic Stress Disorder checklist
PHQ: Patient Health Questionnaire
PMIE: Potentially Morally Injurious Event
PPE: Personal Protective Equipment
PPI: Patient and Public Involvement
PTGI-SF: Post-Traumatic Growth Inventory Short Form
PTSD: Post-Traumatic Stress Disorder
SD: Standard Deviation
UHLS: United Kingdom Household Longitudinal Survey
WEMWBS: Warwick Edinburgh Mental Wellbeing Scale
