# Supplementary material for: Mental health of healthcare workers in England during the first three years of the COVID-19 pandemic: The NHS CHECK study cohort
Source: PLoS One. 2026 Jun 26;21(6):e0350918. doi: 10.1371/journal.pone.0350918 (PMC13309052; doi:10.1371/journal.pone.0350918)
Supplement: S1 File — (DOCX) [file pone.0350918.s001.docx]

# Supplementary file 1 - Job roles

Non-clinical roles were categorised as:

- Administrative and Clerical
- Catering Services
- Chaplaincy
- Clinical Support
- Domestic Services
- Estates Services
- Finance
- Healthcare Scientists (laboratory, technician)
- Human Resources
- IT Support
- Management
- Research / Academic
- Social Services (social worker)
- Support Services (driver, porter, security, stores)

Clinical roles were categorised as:

- Dietitian
- Doctor
- Healthcare Assistant/Nursing Assistant
- Healthcare Scientist (laboratory, technician)
- Medical Associate/Assistant Professions
- Midwife
- Nurse
- Occupational Therapist
- Paramedic/Ambulance Service
- Pharmacist/Pharmacy Technician
- Physiotherapist
- Psychologist/Assistant Psychologist
- Radiographer
- Speech & Language Therapist
- Ward Manager

# Supplementary file 2 – Tables

Table 1 Measures used at each time point

| **Measure** | **Baseline** | | **Six-months** | | **Twelve-months** | | **Thirty-two-months** |
| --- | --- | --- | --- | --- | --- | --- | --- |
|  | Short survey | Long survey | Short survey | Long survey | Short survey | Long survey |  |
| GHQ-12 | ✓ |  | ✓ |  | ✓ |  | ✓ |
| GAD-7 |  | ✓ | ✓ |  | ✓ |  | ✓ |
| PHQ-9 |  | ✓ | ✓ |  | ✓ |  | ✓ |
| AUDIT-C |  | ✓ |  | ✓ | ✓ |  | ✓ |
| PCL-6 |  | ✓ |  | ✓ | ✓ |  | ✓ |
| BAT-12 |  | ✓ |  | ✓ |  | ✓ | ✓ |
| WEMWBS |  | ✓ |  | ✓ |  | ✓ | ✓ |
| PTGI |  |  | ✓ |  | ✓ |  | ✓ |
| MIES |  | ✓ | ✓ |  | ✓ |  | ✓ |
| Age | ✓ |  |  |  |  |  |  |
| Sex | ✓ |  |  |  |  |  |  |
| Ethnicity | ✓ |  |  |  |  |  |  |
| Relationship status | ✓ |  |  |  |  |  |  |
| Job role | ✓ |  |  |  |  |  |  |
| Job setting | ✓ |  |  |  |  |  |  |
| Redeployed | ✓ |  |  |  |  |  |  |
| PPE access | ✓ |  |  |  |  |  |  |
| Felt supported by colleagues | ✓ |  | ✓ |  | ✓ |  | ✓ |
| Felt supported by manager | ✓ |  | ✓ |  | ✓ |  | ✓ |
| Pay grade | ✓ |  |  |  |  |  |  |

Table 2 Attrition analyses - differences between responders and non-responders at each time point

| **Continuous variables (means)** | | | | | |
| --- | --- | --- | --- | --- | --- |
| **Did 6 month survey** | **Age** | **Difference in means** | **95% CI** | **p value** |  |
| No | 39.35 | 3.46 | 3.08 to 3.84 | <0.001 |  |
| Yes | 42.81 |  |  |  |  |
|  |  |  |  |  |  |
| **Did 12 month survey** |  |  |  |  |  |
| No | 38.34 | 4.39 | 4.01 to 4.76 | <0.001 |  |
| Yes | 42.72 |  |  |  |  |
|  |  |  |  |  |  |
| **Did 32 month survey** |  |  |  |  |  |
| No | 38.34 | 5.02 | 4.58 to 5.46 | <0.001 |  |
| Yes | 43.35 |  |  |  |  |
|  |  |  |  |  |  |
| **Categorical variables (proportions)** | | | | | |
| **Sex** | | | | | |
| **Did 6 month survey** | **Female** | **Male** |  |  |  |
| No | 0.54 | 0.56 |  |  |  |
| Yes | 0.46 | 0.44 |  |  |  |
|  |  |  |  |  | p = 0.022 |
| **Did 12 month survey** |  |  |  |  |  |
| No | 0.49 | 0.50 |  |  |  |
| Yes | 0.51 | 0.50 |  |  |  |
|  |  |  |  |  | p = 0.161 |
| **Did 32 month survey** |  |  |  |  |  |
| No | 0.71 | 0.71 |  |  |  |
| Yes | 0.28 | 0.29 |  |  |  |
|  |  |  |  |  | p = 0.400 |
| **Ethnicity** | | | | | |
| **Did 6 month survey** | **White** | **Black** | **Asian** | **Mixed** | **Other** |
| No | 0.52 | 0.70 | 0.64 | 0.61 | 0.63 |
| Yes | 0.48 | 0.30 | 0.36 | 0.39 | 0.37 |
|  |  |  |  |  | p = <0.001 |
| **Did 12 month survey** |  |  |  |  |  |
| No | 0.46 | 0.63 | 63 | 52 | 61 |
| Yes | 0.54 | 0.37 | 37 | 48 | 39 |
|  |  |  |  |  | p = <0.001 |
| **Did 32 month survey** |  |  |  |  |  |
| No | 0.70 | 0.82 | 0.79 | 0.76 | 0.73 |
| Yes | 0.30 | 0.18 | 0.21 | 0.24 | 0.27 |
|  |  |  |  |  | p = <0.001 |
| **Job role** | | | | | |
| **Did 6 month survey** | **Doctor** | **Nurse** | **Other Clinical** | **Non Clinical** |  |
| No | 0.56 | 0.56 | 0.57 | 0.50 |  |
| Yes | 0.44 | 0.44 | 0.43 | 0.50 |  |
|  |  |  |  |  | p = <0.001 |
| **Did 12 month survey** |  |  |  |  |  |
| No | 0.54 | 0.51 | 0.51 | 0.45 |  |
| Yes | 0.46 | 0.49 | 0.49 | 0.55 |  |
|  |  |  |  |  | p = <0.001 |
| **Did 32 month survey** |  |  |  |  |  |
| No | 0.76 | 0.73 | 0.74 | 0.69 |  |
| Yes | 0.24 | 0.27 | 0.26 | 0.31 |  |
|  |  |  |  |  | p = <0.001 |

Table 3 Prevalence of adverse mental health outcomes at each time point

|  | **Baseline** | | | **6 months** | | | **12 months** | | | **32 months** | | |
| --- | --- | --- | --- | --- | --- | --- | --- | --- | --- | --- | --- | --- |
|  | n/N  meeting cut-off score | % meeting cut-off score (95%CI) | Mean (95%CI) | n/N meeting cut-off score | % meeting cut-off score (95%CI) | Mean (95%CI) | n/N meeting cut-off score | % meeting cut-off score (95%CI) | Mean (95%CI) | n/N meeting cut-off score | % meeting cut-off score (95%CI) | Mean (95%CI) |
| **Common mental disorders**  *(GHQ-12: Cut-off ≥ 4, range: 0-12)* | 10,694/  20,694 | 52 (51, 53) | 4.5 (4.4, 4.5) | 4,745/  9,398 | 51 (50, 53) | 4.5 (4.4, 4.6) | 5,170/ 11,110 | 47 (46, 49) | 4.2 (4.1, 4.3) | 3,237/  6,577 | 50 (48, 52) | 4.6 (4.4, 4.7) |
| **Anxiety**  *(GAD-7: Cut-off ≥ 10, range: 0-28)* | 2,627/  11,743 | 23 (22, 24) | 6.4 (6.2,6.5) | 2,069/  9,418 | 24 (22, 25) | 6.2 (6.0, 6.3) | 2,345/ 10,988 | 23 (22, 24) | 6.1 (6.0, 6.3) | 1,281/  6,199 | 21 (19, 22) | 5.8 (5.6, 6.0) |
| **Depression**  *(PHQ-9: Cut-off ≥ 10, range 0-36* | 3,204/  11,710 | 28 (27, 29) | 7.2 (7.0, 7.3) | 2,399/  9,370 | 27 (26, 28) | 6.9 (6.7, 7.1) | 2,948/ 10,925 | 29 (27, 30) | 7.0 (6.8, 7.2) | 1,580/  6,192 | 26 (24,28) | 6.6 (6.4, 6.9) |
| **Alcohol misuse**  *(AUDIT: Cut-off ≥8, range 0-45* | 1,362/  11,132 | 11 (11, 12) | 3.6 (3.5, 3.7) | 1,826/  9,204 | 18 (17, 19) | 4.6 (4.5, 4.7) | 1,452/ 10,843 | 13 (12,14) | 3.7 (3.6, 3.8) | 691/  6,209 | 10 (9, 12) | 3.3 (3.1, 3.5) |
| **PTSD**  *(PCL-6: Cut-off ≥14, range 6-30)* | 2,738/  11,696 | 25 (24, 26) | 11.1 (11.0, 11.3) | 3,257/  9,572 | 37 (36, 38) | 12.7 (12.5, 12.8) | 2,904/  11,252 | 28 (27, 30) | 11.6 (11.5, 11.8) | 1,728/  6,231 | 30 (28, 32) | 11.7 (11.5, 11.9) |
| **Burnout** *(BAT: Cut-off ≥2.96, range 1-5)* | 1,768/  11,535 | 16 (15, 17) | 2.3 (2.2, 2.3) | 887/  4,379 | 21 (19, 23) | 2.4 (2.4, 2.5) | 1,431/  6,156 | 26 (24, 27) | 2.5 (2.5, 2.5) | 1,084/  5,713 | 19 (18, 21) | 2.4 (2.3, 2.4) |
| **Wellbeing**  *(WEMWBS: Cut-off ≥42, range 14-70)* | 6,307/  11,746 | 53 (52, 54) | 43.5 (43.3, 43.7) | 2,381/  4,531 | 51 (49, 53) | 43.4 (42.9, 43.9) | 3,319/  6,211 | 52 (50, 53) | 43.6 (43.1, 44.0) | 3,371/  6,211 | 52 (50, 54) | 43.8 (43.4, 44.3) |
| **Resilience**  *(BRS: Cut-off ≥24,*  *range 6-30)* | 3,511/  11,575 | 30 (29, 31) | 20.4 (20.3, 20.6) | 1,485/  4,551 | 33 (31, 35) | 29.0 (29.0, 29.0) | 2,098/  6,217 | 33 (32, 35) | 29.0 (29.0, 29.0) | Not collected at 32 months | | |
| **Post-Traumatic Growth**  *(PTGI : Cut-off* *≥17, range 0-50)* | Not collected at baseline | | | 2,812/  8,974 | 34 (33, 36) | 13.4 (13.0, 13.7) | 3,209/  10,222 | 35 (33, 36) | 12.7 (12.4, 13.1) | 1,935/  5,899 | 37 (35, 39) | 12.5 (12.0, 12.9) |

Table 4 Unadjusted logistic regression models

|  | **Baseline**  OR (95%CI) p≤ | **6 months**  OR (95%CI) p≤ | **12 months**  OR (95%CI) p≤ | **32 months**  OR (95%CI) p≤ |
| --- | --- | --- | --- | --- |
| GHQ | 1.08 [1.02, 1.16] 0.02 | 1.00 [0.87, 1.12] 0.82 | 0.89 [0.83, 0.96] <0.01 | 1.00 [0.91, 1.12] 0.94 |
| GAD-7 | 0.29 [0.26, 0.32] <0.01 | 0.29 [0.25, 0.33] <0.01 | 0.29 [0.26, 0.32] <0.01 | 0.26 [0.24, 0.28] <0.01 |
| PHQ-9 | 0.38 [0.34, 0.42] <0.01 | 0.35 [0.31, 0.40] <0.01 | 0.39 [0.35, 0.43] <0.01 | 0.35 [0.31, 0.39] <0.01 |
| AUDIT | 0.13 [0.11, 0.15] <0.01 | 0.23 [0.21, 0.29] <0.01 | 0.15 [0.12, 0.17] <0.01 | 0.11 [0.10, 0.13] <0.01 |
| PCL-6 | 0.30 [0.26, 0.34] <0.01 | 0.52 [0.43, 0.62] <0.01 | 0.36 [0.32, 0.41] <0.01 | 0.42 [0.37, 0.47] <0.01 |
| BAT | 0.18 [0.16, 0.21] <0.01 | 0.24 [0.21, 0.29] <0.01 | 0.31 [0.29, 0.35] <0.01 | 0.22 [0.20, 0.27] <0.01 |
| WEMWBS | 1.16 [1.07, 1.26] <0.01 | 1.02 [0.60, 1.72] 0.07 | 1.13 [1.01, 1.27] 0.04 | 1.14 [1.01, 1.27] 0.03 |
| BRS | 0.43 [0.40, 0.46] <0.01 | 0.47 [0.42, 0.53] <0.01 | 0.49 [0.44, 0.55] <0.01 | - |
| PTGI | - | 0.49 [0.44, 0.55] <0.01 | 0.50 [0.46, 0.55] <0.01 | 0.56 [0.50, 0.63] <0.01 |

## Regression analyses – secondary outcome variables

Table 5 GAD-7 regression analyses

|  | **Baseline**  AOR (95%CI) p≤ | | | **6 months**  AOR (95%CI) p≤ | | | **12 months**  AOR (95%CI) p≤ | | | **32 months**  AOR (95%CI) p≤ | | |
| --- | --- | --- | --- | --- | --- | --- | --- | --- | --- | --- | --- | --- |
| **Age (ref: ≤30)** | | | | | | | | | | | | |
| 31-40 | 0.83 | (0.67, 1.01) | 0.06 | 0.97 | (0.68, 1.36) | 0.83 | 1.27 | (0.91, 1.77) | 0.15 | 0.97 | (0.67, 1.42) | 0.89 |
| 41-50 | 0.61 | (0.47, 0.81) | **<0.01** | 0.58 | (0.38, 0.89) | **0.02** | 0.72 | (0.56, 0.93) | **0.01** | 0.87 | (0.65, 1.15) | 0.31 |
| 51-60 | 0.52 | (0.43, 0.63) | **<0.01** | 0.48 | (0.35, 0.67) | **<0.01** | 0.80 | (0.57, 1.13) | 0.18 | 0.72 | (0.49, 1.05) | 0.09 |
| ≥61 | 0.42 | (0.28, 0.63) | **<0.01** | 0.47 | (0.30, 0.74) | **<0.01** | 0.69 | (0.41, 1.17) | 0.16 | 0.37 | (0.16, 0.87) | **0.03** |
| **Sex (ref: Female)** | | | | | | | | | | | | |
| Male | 0.68 | (0.52, 0.89) | **0.01** | 1.31 | (0.92, 1.87) | 0.12 | 0.90 | (0.58, 1.39) | 0.61 | 0.83 | (0.57, 1.21) | 0.31 |
| **Ethnicity (ref: White)** | | | | | | | | | | | | |
| Black | 0.96 | (0.57, 1.63) | 0.89 | 0.61 | (0.25, 1.49) | 0.26 | 0.73 | (0.33, 1.60) | 0.41 | 0.89 | (0.32, 2.51) | 0.82 |
| Asian | 0.61 | (0.39, 0.94) | **0.03** | 0.74 | (0.55, 1.00) | **0.05** | 1.45 | (0.70, 3.02) | 0.30 | 0.33 | (0.10, 1.12) | 0.07 |
| Mixed | 1.61 | (0.65, 4.02) | 0.28 | 2.21 | (1.04, 4.73) | **0.04** | 1.39 | (0.50, 3.89) | 0.51 | 2.48 | (0.77, 7.95) | 0.12 |
| Other | 0.94 | (0.28, 3.17) | 0.91 | 1.82 | (0.70, 4.71) | 0.20 | 1.43 | (0.38, 5.36) | 0.58 | 1.06 | (0.23, 4.86) | 0.93 |
| **Relationship (ref: Single)** | | | | | | | | | | | | |
| In a relationship | 0.87 | (0.76, 0.98) | **0.03** | 1.01 | (0.67, 1.54) | 0.94 | 0.98 | (0.80, 1.20) | 0.82 | 0.65 | (0.45, 0.93) | **0.02** |
| **Job role (ref: Non-clinical)** | | | | | | | | | | | | |
| Doctor | 0.88 | (0.22, 3.43) | 0.84 | 0.41 | (0.08, 2.11) | 0.26 | 0.23 | (0.02, 2.69) | 0.23 | 0.04 | (0.00, 0.47) | **0.01** |
| Nurse | 1.17 | (0.89, 1.52) | 0.24 | 0.90 | (0.70, 1.16) | 0.38 | 1.17 | (0.95, 1.45) | 0.13 | 0.84 | (0.62, 1.14) | 0.24 |
| Other clinical | 0.88 | (0.68, 1.13) | 0.29 | 1.05 | (0.89, 1.24) | 0.56 | 1.13 | (0.82, 1.56) | 0.43 | 1.15 | (0.88, 1.50) | 0.28 |
| **Pay grade (ref: AfC 5 and below)** | | | | | | | | | | | | |
| AfC 6 and above | 0.70 | (0.55, 0.88) | **0.01** | 0.92 | (0.67, 1.28) | 0.60 | 0.77 | (0.49, 1.22) | 0.25 | 0.80 | (0.52, 1.22) | 0.28 |
| **Setting (ref: Other hospital)** | | | | | | | | | | | | |
| Accident & Emergency | 0.83 | (0.51, 1.34) | 0.41 | 2.07 | (0.67, 6.35) | 0.19 | 0.50 | (0.30, 0.84) | **0.01** | 1.95 | (0.81, 4.67) | 0.13 |
| ICU/Critical Care | 1.19 | (0.83, 1.71) | 0.31 | 1.04 | (0.69, 1.57) | 0.85 | 0.89 | (0.52, 1.54) | 0.67 | 1.76 | (0.99, 3.11) | 0.05 |
| Community | 0.91 | (0.76, 1.10) | 0.30 | 1.00 | (0.77, 1.30) | 1.00 | 0.84 | (0.68, 1.04) | 0.10 | 1.16 | (0.89, 1.51) | 0.26 |
| Non-patient-facing | 0.77 | (0.59, 1.00) | **0.05** | 0.68 | (0.42, 1.11) | 0.12 | 0.68 | (0.43, 1.09) | 0.10 | 0.87 | (0.48, 1.61) | 0.65 |
| **PPE (ref: Never/ Sometimes)** | | | | | | | | | | | | |
| Often/Always | 0.84 | (0.64, 1.10) | 0.19 | 0.80 | (0.55, 1.16) | 0.22 | 0.89 | (0.59, 1.32) | 0.53 | 0.55 | (0.35, 0.85) | **0.01** |
| **Colleague support (ref: Not at all/A little)** | | | | | | | | | | | | |
| Moderately/Extremely | 0.69 | (0.47, 1.01) | 0.06 | 0.46 | (0.26, 0.82) | **0.01** | 0.42 | (0.29, 0.61) | **<0.01** | 1.19 | (0.72, 1.98) | 0.47 |
| **Manager support (ref: Not at all/A little)** | | | | | | | | | | | | |
| Moderately/Extremely | 0.62 | (0.55, 0.70) | **<0.01** | 0.99 | (0.69, 1.42) | 0.94 | 1.00 | (0.70, 1.42) | 1.00 | 1.33 | (0.81, 2.19) | 0.24 |
| **Moral injury (ref: below cut-off)** | | | | | | | | | | | | |
| Met cut-off | 2.46 | (2.18, 2.78) | **<0.01** | 1.31 | (1.05, 1.64) | **0.02** | 1.40 | (1.17, 1.67) | **<0.01** | 1.43 | (0.89, 2.29) | 0.13 |
| **Burden period (ref: Time 2 – low pressure)** | | | | | | | | | | | | |
| T1 | 1.04 | (0.84, 1.29) | 0.68 | 1.30 | (1.00, 1.69) | **0.05** | 0.86 | (0.64, 1.16) | 0.31 | 0.80 | (0.47, 1.37) | 0.39 |
| T3 | 1.33 | (1.07, 1.66) | **0.01** | 0.84 | (0.60, 1.18) | 0.30 | 1.14 | (0.90, 1.43) | 0.26 | 0.80 | (0.55, 1.15) | 0.20 |
| **GAD (ref: below cut-off)** | | | | | | | | | | | | |
| Met GAD baseline cut-off | - | - | - | 7.16 | (5.69, 8.99) | **<0.01** | 5.92 | (4.63, 7.58) | **<0.01** | 5.83 | (4.18, 8.13) | **<0.01** |

Table 6 PHQ-9 regression analyses

|  | **Baseline**  AOR (95%CI) p≤ | | | **6 months**  AOR (95%CI) p≤ | | | **12 months**  AOR (95%CI) p≤ | | | **32 months**  AOR (95%CI) p≤ | | |
| --- | --- | --- | --- | --- | --- | --- | --- | --- | --- | --- | --- | --- |
| **Age (ref: ≤30)** | | | | | | | | | | | | |
| 31-40 | 0.92 | (0.78, 1.09) | 0.30 | 1.12 | (0.76, 1.65) | 0.55 | 0.94 | (0.77, 1.14) | 0.49 | 0.84 | (0.49, 1.43) | 0.49 |
| 41-50 | 0.68 | (0.52, 0.90) | **0.01** | 0.65 | (0.46, 0.91) | **0.02** | 0.70 | (0.54, 0.90) | **0.01** | 0.88 | (0.59, 1.32) | 0.52 |
| 51-60 | 0.63 | (0.49, 0.81) | **<0.01** | 0.55 | (0.43, 0.71) | **<0.01** | 0.65 | (0.55, 0.76) | **<0.01** | 0.56 | (0.36, 0.88) | **0.02** |
| ≥61 | 0.55 | (0.40, 0.76) | **<0.01** | 0.43 | (0.28, 0.65) | **<0.01** | 0.64 | (0.44, 0.94) | **0.03** | 0.32 | (0.17, 0.58) | **<0.01** |
| **Sex (ref: Female)** | | | | | | | | | | | | |
| Male | 0.83 | (0.66, 1.04) | 0.09 | 1.04 | (0.78, 1.39) | 0.80 | 0.91 | (0.70, 1.18) | 0.44 | 1.03 | (0.74, 1.45) | 0.85 |
| **Ethnicity (ref: White)** | | | | | | | | | | | | |
| Black | 0.83 | (0.43, 1.59) | 0.55 | 0.52 | (0.28, 0.96) | **0.04** | 0.60 | (0.29, 1.24) | 0.15 | 0.75 | (0.37, 1.54) | 0.41 |
| Asian | 0.79 | (0.53, 1.19) | 0.24 | 0.83 | (0.61, 1.13) | 0.22 | 1.32 | (0.66, 2.65) | 0.41 | 0.40 | (0.22, 0.75) | 0.01 |
| Mixed | 0.96 | (0.62, 1.49) | 0.84 | 1.27 | (0.71, 2.28) | 0.40 | 0.74 | (0.44, 1.25) | 0.24 | 2.64 | (1.01, 6.87) | 0.05 |
| Other | 1.32 | (0.74, 2.35) | 0.32 | 0.77 | (0.19, 3.06) | 0.69 | 1.43 | (0.37, 5.61) | 0.59 | 0.81 | (0.26, 2.54) | 0.71 |
| **Relationship (ref: Single)** | | | | | | | | | | | | |
| In a relationship | 0.72 | (0.62, 0.83) | **<0.01** | 0.86 | (0.61, 1.20) | 0.34 | 0.92 | (0.76, 1.12) | 0.38 | 0.58 | (0.42, 0.79) | **<0.01** |
| **Job role (ref: Non-clinical)** | | | | | | | | | | | | |
| Doctor | 0.42 | (0.08, 2.11) | 0.27 | 0.50 | (0.10, 2.57) | 0.38 | 0.72 | (0.11, 4.62) | 0.72 | 0.60 | (0.03, 12.06) | 0.73 |
| Nurse | 1.14 | (0.91, 1.42) | 0.23 | 1.14 | (0.83, 1.57) | 0.39 | 1.53 | (1.22, 1.91) | **<0.01** | 0.95 | (0.69, 1.31) | 0.76 |
| Other clinical | 0.95 | (0.76, 1.18) | 0.62 | 0.98 | (0.78, 1.23) | 0.88 | 1.28 | (0.90, 1.84) | 0.16 | 1.14 | (0.90, 1.45) | 0.25 |
| **Pay grade (ref: AfC 5 and below)** | | | | | | | | | | | | |
| AfC 6 and above | 0.66 | (0.56, 0.77) | **<0.01** | 0.81 | (0.59, 1.12) | 0.19 | 0.70 | (0.49, 0.99) | **0.05** | 0.75 | (0.52, 1.08) | 0.11 |
| **Setting (ref: Other hospital)** | | | | | | | | | | | | |
| Accident & Emergency | 0.82 | (0.37, 1.83) | 0.61 | 2.12 | (1.07, 4.19) | **0.03** | 3.42 | (1.64, 7.12) | **<0.01** | 1.91 | (0.77, 4.76) | 0.15 |
| ICU/Critical Care | 1.36 | (0.90, 2.08) | 0.14 | 1.19 | (0.77, 1.84) | 0.41 | 1.09 | (0.71, 1.69) | 0.68 | 2.17 | (1.09, 4.32) | **0.03** |
| Community | 0.95 | (0.78, 1.16) | 0.58 | 1.02 | (0.69, 1.50) | 0.94 | 0.76 | (0.60, 0.96) | **0.02** | 1.09 | (0.87, 1.37) | 0.41 |
| Non-patient-facing | 1.07 | (0.72, 1.59) | 0.73 | 0.83 | (0.44, 1.56) | 0.53 | 0.68 | (0.38, 1.25) | 0.20 | 0.78 | (0.39, 1.56) | 0.46 |
| **PPE (ref: Never/ Sometimes)** | | | | | | | | | | | | |
| Often/Always | 0.73 | (0.58, 0.92) | **0.01** | 0.87 | (0.63, 1.21) | 0.39 | 0.64 | (0.42, 0.97) | **0.04** | 0.50 | (0.34, 0.72) | **<0.01** |
| **Colleague support (ref: Not at all/A little)** | | | | | | | | | | | | |
| Moderately/Extremely | 0.60 | (0.46, 0.78) | **<0.01** | 0.60 | (0.36, 0.98) | **0.04** | 0.51 | (0.34, 0.76) | <0.01 | 0.99 | (0.52, 1.86) | 0.97 |
| **Manager support (ref: Not at all/A little)** | | | | | | | | | | | | |
| Moderately/Extremely | 0.64 | (0.54, 0.77) | **<0.01** | 0.93 | (0.72, 1.20) | 0.55 | 0.93 | (0.76, 1.13) | 0.45 | 1.22 | (0.76, 1.95) | 0.38 |
| **Moral injury (ref: below cut-off)** | | | | | | | | | | | | |
| Met cut-off | 2.09 | (1.82, 2.41) | **<0.01** | 1.45 | (1.13, 1.85) | **0.01** | 1.30 | (1.09, 1.55) | **0.01** | 1.49 | (1.12, 1.97) | **0.01** |
| **Burden period (ref: Time 2 – low pressure)** | | | | | | | | | | | | |
| T1 | 1.12 | (1.02, 1.23) | **0.02** | 1.37 | (1.07, 1.76) | **0.02** | 0.75 | (0.55, 1.03) | 0.07 | 0.99 | (0.49, 2.02) | 0.98 |
| T3 | 1.27 | (1.03, 1.56) | **0.03** | 0.92 | (0.68, 1.25) | 0.58 | 1.17 | (0.92, 1.47) | 0.18 | 0.90 | (0.69, 1.17) | 0.39 |
| **PHQ (ref: below cut-off)** | | | | | | | | | | | | |
| Met PHQ baseline cut-off | - | - | - | 7.86 | (6.14, 10.07) | **<0.01** | 6.49 | (4.35, 9.69) | **<0.01** | 5.70 | (4.03, 8.08) | **<0.01** |

Table 7 AUDIT regression analyses

|  | **Baseline**  AOR (95%CI) p≤ | | | **6 months**  AOR (95%CI) p≤ | | | **12 months**  AOR (95%CI) p≤ | | | **32 months**  AOR (95%CI) p≤ | | |
| --- | --- | --- | --- | --- | --- | --- | --- | --- | --- | --- | --- | --- |
| **Age (ref: ≤30)** | | | | | | | | | | | | |
| 31-40 | 0.88 | (0.75, 1.02) | 0.09 | 0.88 | (0.61, 1.28) | 0.49 | 0.88 | (0.54, 1.45) | 0.60 | 1.24 | (0.47, 3.27) | 0.65 |
| 41-50 | 1.35 | (1.01, 1.80) | **0.04** | 0.87 | (0.58, 1.30) | 0.47 | 0.74 | (0.47, 1.19) | 0.20 | 1.32 | (0.85, 2.03) | 0.20 |
| 51-60 | 1.05 | (0.78, 1.42) | 0.72 | 0.81 | (0.55, 1.19) | 0.27 | 0.76 | (0.43, 1.34) | 0.32 | 0.92 | (0.41, 2.08) | 0.84 |
| ≥61 | 0.77 | (0.57, 1.04) | 0.08 | 0.95 | (0.42, 2.15) | 0.90 | 0.58 | (0.23, 1.46) | 0.23 | 1.26 | (0.51, 3.14) | 0.60 |
| **Sex (ref: Female)** | | | | | | | | | | | | |
| Male | 1.65 | (1.36, 2.00) | **<0.01** | 1.42 | (0.95, 2.14) | 0.08 | 1.68 | (1.01, 2.79) | **0.05** | 1.30 | (0.72, 2.35) | 0.36 |
| **Ethnicity (ref: White)** | | | | | | | | | | | | |
| Black | 0.28 | (0.11, 0.70) | **0.01** | 0.39 | (0.10, 1.56) | 0.17 | 0.54 | (0.13, 2.26) | 0.37 | 0.13 | (0.02, 0.85) | **0.04** |
| Asian | 0.22 | (0.10, 0.48) | **<0.01** | 0.21 | (0.11, 0.40) | **<0.01** | 0.85 | (0.35, 2.07) | 0.71 | 0.83 | (0.24, 2.93) | 0.76 |
| Mixed | 0.53 | (0.38, 0.75) | **<0.01** | 1.16 | (0.37, 3.67) | 0.78 | 4.56 | (0.62, 33.72) | 0.13 | 0.49 | (0.10, 2.37) | 0.36 |
| Other | 0.33 | (0.08, 1.37) | 0.12 | 0.26 | (0.04, 1.81) | 0.16 | 3.71 | (0.55, 24.96) | 0.16 | Insufficient sample size | | |
| **Relationship (ref: Single)** | | | | | | | | | | | | |
| In a relationship | 0.88 | (0.74, 1.05) | 0.14 | 1.25 | (0.97, 1.60) | 0.08 | 0.95 | (0.73, 1.24) | 0.67 | 0.88 | (0.45, 1.69) | 0.67 |
| **Job role (ref: Non-clinical)** | | | | | | | | | | | | |
| Doctor | 0.97 | (0.26, 3.58) | 0.96 | 0.12 | (0.02, 0.62) | **0.02** | 0.36 | (0.08, 1.58) | 0.16 | 0.03 | (0.00, 0.68) | **0.03** |
| Nurse | 1.06 | (0.81, 1.38) | 0.64 | 0.66 | (0.49, 0.90) | **0.01** | 0.61 | (0.40, 0.91) | **0.02** | 1.10 | (0.56, 2.15) | 0.77 |
| Other clinical | 1.00 | (0.81, 1.24) | 1.00 | 0.61 | (0.43, 0.88) | **0.01** | 0.72 | (0.43, 1.21) | 0.20 | 0.85 | (0.48, 1.52) | 0.57 |
| **Pay grade (ref: AfC 5 and below)** | | | | | | | | | | | | |
| AfC 6 and above | 0.88 | (0.74, 1.05) | 0.15 | 1.57 | (1.10, 2.25) | **0.02** | 1.10 | (0.67, 1.80) | 0.70 | 1.08 | (0.46, 2.50) | 0.85 |
| **Setting (ref: Other hospital)** | | | | | | | | | | | | |
| Accident & Emergency | 1.06 | (0.41, 2.74) | 0.90 | 0.74 | (0.33, 1.64) | 0.43 | 0.48 | (0.17, 1.34) | 0.15 | 2.02 | (0.23, 17.72) | 0.50 |
| ICU/Critical Care | 0.88 | (0.48, 1.61) | 0.65 | 1.12 | (0.71, 1.77) | 0.62 | 1.53 | (1.01, 2.30) | **0.04** | 1.26 | (0.51, 3.14) | 0.60 |
| Community | 1.10 | (0.92, 1.30) | 0.27 | 1.03 | (0.78, 1.37) | 0.83 | 1.11 | (0.78, 1.57) | 0.55 | 1.13 | (0.77, 1.65) | 0.52 |
| Non-patient-facing | 0.89 | (0.50, 1.58) | 0.67 | 0.87 | (0.44, 1.74) | 0.68 | 0.39 | (0.11, 1.45) | 0.15 | 0.95 | (0.49, 1.85) | 0.88 |
| **PPE (ref: Never/ Sometimes)** | | | | | | | | | | | | |
| Often/Always | 1.08 | (0.78, 1.48) | 0.62 | 0.86 | (0.58, 1.28) | 0.42 | 1.38 | (0.74, 2.56) | 0.29 | 0.70 | (0.45, 1.09) | 0.11 |
| **Colleague support (ref: Not at all/A little)** | | | | | | | | | | | | |
| Moderately/Extremely | 1.25 | (0.86, 1.82) | 0.22 | 1.07 | (0.65, 1.78) | 0.77 | 1.23 | (0.75, 2.00) | 0.39 | 0.99 | (0.49, 2.01) | 0.98 |
| **Manager support (ref: Not at all/A little)** | | | | | | | | | | | | |
| Moderately/Extremely | 0.93 | (0.69, 1.25) | 0.62 | 0.82 | (0.50, 1.37) | 0.43 | 0.69 | (0.42, 1.15) | 0.15 | 0.83 | (0.59, 1.18) | 0.28 |
| **Moral injury (ref: below cut-off)** | | | | | | | | | | | | |
| Met cut-off | 1.22 | (0.96, 1.54) | 0.09 | 0.94 | (0.66, 1.33) | 0.70 | 0.98 | (0.67, 1.42) | 0.90 | 0.93 | (0.56, 1.56) | 0.78 |
| **Burden period (ref: Time 2 – low pressure)** | | | | | | | | | | | | |
| T1 | 1.27 | (0.85, 1.92) | 0.23 | 1.07 | (0.75, 1.51) | 0.70 | 1.23 | (0.75, 2.03) | 0.39 | 1.21 | (0.82, 1.78) | 0.31 |
| T3 | 0.95 | (0.79, 1.15) | 0.60 | 2.05 | (1.66, 2.53) | **<0.01** | 1.45 | (0.88, 2.38) | 0.13 | 1.04 | (0.50, 2.13) | 0.92 |
| **AUDIT (ref: below cut-off)** | | | | | | | | | | | | |
| Met AUDIT baseline cut-off | - | - | - | 22.53 | (12.36, 41.07) | **<0.01** | 29.05 | (18.67, 45.20) | **<0.01** | 31.47 | (19.54, 50.68) | **<0.01** |

Table 8 PCL-6 regression analyses

|  | **Baseline**  AOR (95%CI) p≤ | | | **6 months**  AOR (95%CI) p≤ | | | **12 months**  AOR (95%CI) p≤ | | | **32 months**  AOR (95%CI) p≤ | | |
| --- | --- | --- | --- | --- | --- | --- | --- | --- | --- | --- | --- | --- |
| **Age (ref: ≤30)** | | | | | | | | | | | | |
| 31-40 | 0.93 | (0.75, 1.16) | 0.51 | 0.93 | (0.84, 1.40) | 0.52 | 1.46 | (1.01, 2.10) | 0.04 | 1.08 | (0.68, 1.73) | 0.72 |
| 41-50 | 0.76 | (0.60, 0.96) | **0.02** | 0.76 | (0.45, 0.77) | **<0.01** | 1.24 | (0.93, 1.64) | 0.13 | 0.88 | (0.57, 1.37) | 0.56 |
| 51-60 | 0.70 | (0.52, 0.94) | **0.02** | 0.70 | (0.54, 1.02) | 0.06 | 1.14 | (0.78, 1.68) | 0.47 | 0.64 | (0.45, 0.90) | **0.01** |
| ≥61 | 0.65 | (0.42, 1.02) | 0.06 | 0.65 | (0.39, 0.81) | **<0.01** | 0.99 | (0.62, 1.56) | 0.95 | 0.68 | (0.33, 1.40) | 0.27 |
| **Sex (ref: Female)** | | | | | | | | | | | | |
| Male | 0.67 | (0.51, 0.88) | **0.01** | 0.67 | (0.52, 0.98) | **0.04** | 1.02 | (0.68, 1.54) | 0.91 | 0.96 | (0.75, 1.23) | 0.73 |
| **Ethnicity (ref: White)** | | | | | | | | | | | | |
| Black | 1.07 | (0.64, 1.79) | 0.79 | 1.07 | (0.31, 1.98) | 0.58 | 0.82 | (0.52, 1.28) | 0.36 | 0.59 | (0.33, 1.06) | 0.07 |
| Asian | 1.41 | (1.01, 1.97) | **0.04** | 1.41 | (0.55, 2.00) | 0.87 | 1.37 | (0.85, 2.20) | 0.18 | 0.36 | (0.16, 0.78) | **0.01** |
| Mixed | 1.76 | (0.63, 4.91) | 0.26 | 1.76 | (0.54, 1.88) | 0.98 | 1.09 | (0.36, 3.34) | 0.87 | 2.14 | (0.77, 5.97) | 0.14 |
| Other | 1.54 | (0.60, 3.97) | 0.35 | 1.54 | (0.47, 7.37) | 0.35 | 0.80 | (0.17, 3.80) | 0.77 | 1.22 | (0.28, 5.36) | 0.78 |
| **Relationship (ref: Single)** | | | | | | | | | | | | |
| In a relationship | 0.88 | (0.77, 1.02) | 0.08 | 1.00 | (0.76, 1.31) | 0.99 | 0.96 | (0.74, 1.25) | 0.74 | 0.60 | (0.43, 0.84) | **0.01** |
| **Job role (ref: Non-clinical)** | | | | | | | | | | | | |
| Doctor | 0.74 | (0.12, 4.48) | 0.73 | 0.39 | (0.09, 1.62) | 0.18 | 0.36 | (0.05, 2.34) | 0.26 | 0.46 | (0.04, 4.62) | 0.48 |
| Nurse | 1.07 | (0.75, 1.53) | 0.70 | 1.20 | (0.95, 1.53) | 0.12 | 1.68 | (1.26, 2.24) | **<0.01** | 1.01 | (0.74, 1.39) | 0.94 |
| Other clinical | 0.99 | (0.80, 1.23) | 0.91 | 1.00 | (0.80, 1.24) | 0.96 | 1.18 | (0.78, 1.78) | 0.41 | 0.83 | (0.61, 1.15) | 0.25 |
| **Pay grade (ref: AfC 5 and below)** | | | | | | | | | | | | |
| AfC 6 and above | 0.75 | (0.64, 0.88) | **<0.01** | 0.89 | (0.69, 1.16) | 0.36 | 0.75 | (0.55, 1.04) | 0.08 | 0.98 | (0.70, 1.37) | 0.89 |
| **Setting (ref: Other hospital)** | | | | | | | | | | | | |
| Accident & Emergency | 0.83 | (0.48, 1.42) | 0.48 | 0.87 | (0.48, 1.56) | 0.61 | 3.08 | (1.29, 7.32) | **0.01** | 0.76 | (0.36, 1.58) | 0.44 |
| ICU/Critical Care | 1.79 | (1.10, 2.92) | **0.02** | 1.37 | (0.84, 2.23) | 0.19 | 1.76 | (1.08, 2.89) | **0.03** | 1.43 | (0.87, 2.35) | 0.15 |
| Community | 1.13 | (0.94, 1.38) | 0.18 | 0.91 | (0.72, 1.15) | 0.41 | 0.91 | (0.74, 1.13) | 0.38 | 0.92 | (0.55, 1.54) | 0.75 |
| Non-patient-facing | 1.21 | (0.85, 1.70) | 0.27 | 0.85 | (0.53, 1.36) | 0.47 | 0.72 | (0.39, 1.35) | 0.28 | 0.74 | (0.28, 1.94) | 0.51 |
| **PPE (ref: Never/ Sometimes)** | | | | | | | | | | | | |
| Often/Always | 0.72 | (0.55, 0.95) | **0.02** | 1.21 | (0.69, 2.10) | 0.49 | 0.62 | (0.40, 0.95) | **0.03** | 0.66 | (0.43, 1.03) | 0.06 |
| **Colleague support (ref: Not at all/A little)** | | | | | | | | | | | | |
| Moderately/Extremely | 0.82 | (0.54, 1.24) | 0.33 | 0.63 | (0.41, 0.98) | 0.04 | 0.69 | (0.47, 1.01) | 0.06 | 0.49 | (0.25, 0.93) | 0.03 |
| **Manager support (ref: Not at all/A little)** | | | | | | | | | | | | |
| Moderately/Extremely | 0.80 | (0.61, 1.05) | 0.10 | 0.86 | (0.63, 1.18) | 0.33 | 0.83 | (0.68, 1.01) | 0.06 | 0.79 | (0.46, 1.35) | 0.36 |
| **Moral injury (ref: below cut-off)** | | | | | | | | | | | | |
| Met cut-off | 2.88 | (2.25, 3.68) | **<0.01** | 1.87 | (1.46, 2.38) | **<0.01** | 1.16 | (0.88, 1.54) | 0.28 | 1.68 | (1.25, 2.26) | <0.01 |
| **Burden period (ref: Time 2 – low pressure)** | | | | | | | | | | | | |
| T1 | 1.23 | (0.99, 1.54) | 0.06 | 1.09 | (0.73, 1.61) | 0.66 | 0.89 | (0.69, 1.16) | 0.38 | 0.76 | (0.45, 1.28) | 0.28 |
| T3 | 1.27 | (1.01, 1.59) | **0.04** | 0.61 | (0.49, 0.77) | **<0.01** | 1.39 | (1.04, 1.85) | **0.03** | 0.80 | (0.49, 1.28) | 0.32 |
| **PCL (ref: below cut-off)** | | | | | | | | | | | | |
| Met PCL baseline cut-off | - | - | - | 6.05 | (4.50, 8.13) | **<0.01** | 5.47 | (4.53, 6.61) | **<0.01** | 3.25 | (2.62, 4.03) | **<0.01** |

Table 9 BAT regression analyses

|  | **Baseline**  AOR (95%CI) p≤ | | | **6 months**  AOR (95%CI) p≤ | | | **12 months**  AOR (95%CI) p≤ | | | **32 months**  AOR (95%CI) p≤ | | |
| --- | --- | --- | --- | --- | --- | --- | --- | --- | --- | --- | --- | --- |
| **Age (ref: ≤30)** | | | | | | | | | | | | |
| 31-40 | 1.13 | (0.91, 1.40) | 0.24 | 0.73 | (0.45, 1.18) | 0.18 | 0.89 | (0.55, 1.45) | 0.62 | 0.73 | (0.42, 1.27) | 0.25 |
| 41-50 | 0.81 | (0.67, 1.00) | **0.05** | 0.45 | (0.27, 0.76) | **0.01** | 0.65 | (0.49, 0.85) | **<0.01** | 0.71 | (0.38, 1.30) | 0.25 |
| 51-60 | 0.76 | (0.62, 0.94) | **0.01** | 0.51 | (0.38, 0.69) | **<0.01** | 0.68 | (0.45, 1.03) | 0.07 | 0.44 | (0.23, 0.86) | **0.02** |
| ≥61 | 0.49 | (0.33, 0.72) | **<0.01** | 0.40 | (0.21, 0.73) | **0.01** | 0.49 | (0.31, 0.78) | **<0.01** | 0.15 | (0.04, 0.50) | **<0.01** |
| **Sex (ref: Female)** | | | | | | | | | | | | |
| Male | 0.91 | (0.77, 1.07) | 0.23 | 1.18 | (0.74, 1.89) | 0.47 | 1.32 | (0.74, 2.35) | 0.32 | 1.07 | (0.63, 1.80) | 0.79 |
| **Ethnicity (ref: White)** | | | | | | | | | | | | |
| Black | 0.67 | (0.30, 1.52) | 0.32 | 0.44 | (0.13, 1.54) | 0.19 | 0.60 | (0.22, 1.63) | 0.29 | 0.35 | (0.15, 0.84) | **0.02** |
| Asian | 0.81 | (0.56, 1.18) | 0.25 | 0.51 | (0.23, 1.13) | 0.09 | 0.57 | (0.21, 1.56) | 0.25 | 0.20 | (0.09, 0.43) | **<0.01** |
| Mixed | 1.16 | (0.72, 1.86) | 0.53 | 1.57 | (0.74, 3.36) | 0.22 | 0.84 | (0.41, 1.71) | 0.61 | 2.34 | (0.97, 5.65) | 0.06 |
| Other | 0.65 | (0.09, 4.58) | 0.65 | 0.48 | (0.03, 8.61) | 0.59 | 0.35 | (0.07, 1.85) | 0.20 | 1.15 | (0.22, 6.00) | 0.86 |
| **Relationship (ref: Single)** | | | | | | | | | | | | |
| In a relationship | 0.84 | (0.66, 1.08) | 0.17 | 0.99 | (0.70, 1.39) | 0.94 | 0.91 | (0.74, 1.12) | 0.36 | 0.59 | (0.42, 0.83) | **<0.01** |
| **Job role (ref: Non-clinical)** | | | | | | | | | | | | |
| Doctor | 0.87 | (0.16, 4.87) | 0.87 | 0.31 | (0.03, 2.99) | 0.29 | 0.17 | (0.02, 1.24) | 0.08 | 1.00 |  |  |
| Nurse | 1.10 | (0.87, 1.40) | 0.38 | 0.80 | (0.39, 1.65) | 0.53 | 0.83 | (0.54, 1.27) | 0.37 | 1.18 | (0.78, 1.80) | 0.40 |
| Other clinical | 0.93 | (0.78, 1.10) | 0.36 | 0.85 | (0.64, 1.13) | 0.25 | 1.40 | (1.17, 1.69) | **<0.01** | 1.07 | (0.70, 1.63) | 0.76 |
| **Pay grade (ref: AfC 5 and below)** | | | | | | | | | | | | |
| AfC 6 and above | 0.90 | (0.76, 1.08) | 0.24 | 0.97 | (0.63, 1.51) | 0.90 | 1.43 | (0.99, 2.06) | 0.06 | 1.52 | (1.00, 2.29) | **0.05** |
| **Setting (ref: Other hospital)** | | | | | | | | | | | | |
| Accident & Emergency | 0.74 | (0.45, 1.20) | 0.20 | 1.33 | (0.14, 12.70) | 0.79 | 1.77 | (0.33, 9.55) | 0.48 | 0.27 | (0.14, 0.52) | **<0.01** |
| ICU/Critical Care | 1.39 | (0.92, 2.11) | 0.11 | 1.72 | (0.99, 2.98) | **0.05** | 1.32 | (1.03, 1.70) | **0.03** | 0.75 | (0.39, 1.41) | 0.34 |
| Community | 1.00 | (0.84, 1.19) | 0.98 | 0.97 | (0.72, 1.32) | 0.86 | 0.84 | (0.61, 1.17) | 0.28 | 0.65 | (0.44, 0.97) | 0.04 |
| Non-patient-facing | 0.80 | (0.54, 1.17) | 0.23 | 0.60 | (0.24, 1.48) | 0.25 | 0.72 | (0.20, 2.63) | 0.60 | 1.37 | (0.69, 2.70) | 0.34 |
| **PPE (ref: Never/ Sometimes)** | | | | | | | | | | | | |
| Often/ Always | 0.94 | (0.67, 1.30) | 0.67 | 0.68 | (0.31, 1.48) | 0.31 | 0.62 | (0.28, 1.35) | 0.21 | 0.72 | (0.41, 1.26) | 0.23 |
| **Colleague support (ref: Not at all/A little)** | | | | | | | | | | | | |
| Moderately/Extremely | 0.59 | (0.44, 0.80) | **<0.01** | 0.93 | (0.58, 1.50) | 0.76 | 0.63 | (0.42, 0.94) | **0.03** | 0.77 | (0.44, 1.35) | 0.35 |
| **Manager support (ref: Not at all/A little)** | | | | | | | | | | | | |
| Moderately/Extremely | 0.43 | (0.34, 0.54) | **<0.01** | 0.73 | (0.50, 1.05) | 0.08 | 0.48 | (0.35, 0.66) | **<0.01** | 0.76 | (0.50, 1.17) | 0.19 |
| **Moral injury (ref: below cut-off)** | | | | | | | | | | | | |
| Met cut-off | 2.63 | (2.10, 3.28) | **<0.01** | 1.51 | (1.09, 2.09) | **0.02** | 1.25 | (0.87, 1.77) | 0.21 | 1.29 | (1.00, 1.65) | **0.05** |
| **Burden period (ref: Time 2 – low pressure)** | | | | | | | | | | | | |
| T1 | 0.81 | (0.61, 1.09) | 0.15 | 1.28 | (1.04, 1.58) | **0.03** | 1.00 | (0.61, 1.63) | 0.98 | 1.02 | (0.62, 1.66) | 0.95 |
| T3 | 1.32 | (1.02, 1.70) | **0.04** | 2.38 | (1.38, 4.08) | **<0.01** | 1.25 | (0.79, 1.99) | 0.31 | 0.69 | (0.46, 1.03) | 0.07 |
| **BAT (ref: below cut-off)** | | | | | | | | | | | | |
| Met BAT baseline cut-off |  |  |  | 10.43 | (6.40, 16.98) | **<0.01** | 9.79 | (6.23, 15.38) | **<0.01** | 7.90 | (5.55, 11.23) | **<0.01** |
|  |  |  |  |  |  |  |  |  |  |  |  |  |

Table 10 WEMWBS regression analyses

|  | **Baseline**  AOR (95%CI) p≤ | | | **6 months**  AOR (95%CI) p≤ | | | **12 months**  AOR (95%CI) p≤ | | | **32 months**  AOR (95%CI) p≤ | | |
| --- | --- | --- | --- | --- | --- | --- | --- | --- | --- | --- | --- | --- |
| **Age (ref: ≤30)** | | | | | | | | | | | | |
| 31-40 | 0.96 | (0.82, 1.12) | 0.58 | 1.02 | (0.73, 1.42) | 0.90 | 0.82 | (0.60, 1.11) | 0.18 | 1.04 | (0.59, 1.84) | 0.87 |
| 41-50 | 1.44 | (1.19, 1.74) | **<0.01** | 1.65 | (1.25, 2.18) | **<0.01** | 1.28 | (0.97, 1.68) | 0.08 | 1.24 | (0.70, 2.20) | 0.43 |
| 51-60 | 1.86 | (1.50, 2.31) | **<0.01** | 1.99 | (1.39, 2.83) | **<0.01** | 1.27 | (0.88, 1.83) | 0.18 | 1.46 | (1.01, 2.11) | **0.04** |
| ≥61 | 1.85 | (1.47, 2.32) | **<0.01** | 2.36 | (1.28, 4.34) | **0.01** | 1.39 | (0.84, 2.29) | 0.18 | 1.61 | (0.84, 3.10) | 0.14 |
| **Sex (ref: Female)** | | | | | | | | | | | | |
| Male | 1.03 | (0.84, 1.25) | 0.79 | 1.20 | (0.99, 1.45) | 0.06 | 0.81 | (0.47, 1.39) | 0.42 | 0.88 | (0.56, 1.36) | 0.53 |
| **Ethnicity (ref: White)** | | | | | | | | | | | | |
| Black | 1.57 | (1.16, 2.13) | **0.01** | 1.47 | (0.63, 3.40) | 0.35 | 1.58 | (0.36, 6.97) | 0.52 | 1.85 | (1.06, 3.24) | **0.03** |
| Asian | 1.71 | (1.25, 2.34) | **<0.01** | 1.31 | (0.62, 2.80) | 0.45 | 0.80 | (0.41, 1.54) | 0.48 | 1.52 | (0.91, 2.53) | 0.10 |
| Mixed | 1.51 | (1.05, 2.19) | **0.03** | 0.31 | (0.07, 1.42) | 0.12 | 2.11 | (1.16, 3.84) | 0.02 | 0.21 | (0.05, 0.92) | **0.04** |
| Other | 1.87 | (0.68, 5.12) | 0.21 | 1.43 | (0.48, 4.24) | 0.50 | 0.77 | (0.13, 4.69) | 0.76 | 0.60 | (0.29, 1.25) | 0.16 |
| **Relationship (ref: Single)** | | | | | | | | | | | | |
| In a relationship | 1.36 | (1.14, 1.61) | **<0.01** | 1.10 | (0.85, 1.41) | 0.46 | 1.21 | (0.96, 1.52) | 0.10 | 1.78 | (1.39, 2.29) | **<0.01** |
| **Job role (ref: Non-clinical)** | | | | | | | | | | | | |
| Doctor | 1.88 | (0.43, 8.27) | 0.38 | 0.97 | (0.35, 2.64) | 0.94 | 3.62 | (0.63, 20.70) | 0.14 | 12.05 | (1.05, 138.67) | **0.05** |
| Nurse | 1.02 | (0.81, 1.28) | 0.87 | 1.07 | (0.57, 2.02) | 0.82 | 1.09 | (0.85, 1.40) | 0.45 | 1.00 | (0.73, 1.37) | 1.00 |
| Other clinical | 1.11 | (0.94, 1.31) | 0.22 | 1.15 | (0.58, 2.28) | 0.67 | 0.94 | (0.69, 1.28) | 0.68 | 1.12 | (0.85, 1.47) | 0.39 |
| **Pay grade (ref: AfC 5 and below)** | | | | | | | | | | | | |
| AfC 6 and above | 1.34 | (1.17, 1.54) | **<0.01** | 1.17 | (0.89, 1.54) | 0.23 | 0.79 | (0.54, 1.16) | 0.21 | 1.01 | (0.81, 1.26) | 0.96 |
| **Setting (ref: Other hospital)** | | | | | | | | | | | | |
| Accident & Emergency | 1.13 | (0.54, 2.36) | 0.74 | 0.66 | (0.14, 3.17) | 0.58 | 0.61 | (0.25, 1.51) | 0.27 | 0.59 | (0.22, 1.57) | 0.27 |
| ICU/Critical Care | 0.70 | (0.50, 0.98) | 0.04 | 0.71 | (0.43, 1.18) | 0.17 | 1.32 | (0.73, 2.39) | 0.33 | 0.63 | (0.35, 1.13) | 0.11 |
| Community | 1.10 | (0.94, 1.29) | 0.20 | 1.24 | (0.86, 1.79) | 0.23 | 1.31 | (0.99, 1.72) | **0.05** | 1.01 | (0.77, 1.33) | 0.92 |
| Non-patient-facing | 1.20 | (0.95, 1.51) | 0.12 | 1.36 | (0.63, 2.90) | 0.41 | 1.37 | (0.83, 2.25) | 0.20 | 0.61 | (0.39, 0.95) | **0.03** |
| **PPE (ref: Never/ Sometimes)** | | | | | | | | | | | | |
| Often/ Always | 1.33 | (1.09, 1.62) | **0.01** | 1.17 | (0.62, 2.18) | 0.61 | 1.01 | (0.51, 2.00) | 0.97 | 2.51 | (1.52, 4.13) | **<0.01** |
| **Colleague support (ref: Not at all/A little)** | | | | | | | | | | | | |
| Moderately/Extremely | 2.04 | (1.41, 2.95) | **<0.01** | 1.23 | (0.58, 2.62) | 0.57 | 1.11 | (0.75, 1.65) | 0.58 | 0.80 | (0.44, 1.47) | 0.45 |
| **Manager support (ref: Not at all/A little)** | | | | | | | | | | | | |
| Moderately/Extremely | 2.16 | (1.67, 2.79) | **<0.01** | 1.06 | (0.72, 1.56) | 0.76 | 1.42 | (0.98, 2.07) | 0.06 | 1.14 | (0.86, 1.50) | 0.34 |
| **Moral injury (ref: below cut-off)** | | | | | | | | | | | | |
| Met cut-off | 0.62 | (0.49, 0.77) | **<0.01** | 0.72 | (0.57, 0.91) | **0.01** | 0.71 | (0.53, 0.97) | **0.03** | 0.77 | (0.59, 0.99) | **0.04** |
| **Burden period (ref: Time 2 – low pressure)** | | | | | | | | | | | | |
| T1 | 1.22 | (1.08, 1.39) | **<0.01** | 0.80 | (0.61, 1.05) | 0.11 | 0.87 | (0.61, 1.24) | 0.43 | 1.40 | (0.77, 2.56) | 0.25 |
| T3 | 0.83 | (0.74, 0.93) | **<0.01** | 1.25 | (0.57, 2.74) | 0.55 | 0.62 | (0.44, 0.87) | **0.01** | 1.33 | (0.95, 1.85) | 0.09 |
| **WEMWBS (ref: below cut-off)** | | | | | | | | | | | | |
| Met WEMWBS baseline cut-off |  |  |  | 8.47 | (6.94, 10.33) | **<0.01** | 5.80 | (4.74, 7.10) | **<0.01** | 5.23 | (4.14, 6.60) | **<0.01** |
|  |  |  |  |  |  |  |  |  |  |  |  |  |

Table 11 BRS regression analyses

|  | **Baseline**  AOR (95%CI) p≤ | | | | **6 months**  AOR (95%CI) p≤ | | | | **12 months**  AOR (95%CI) p≤ | | | |
| --- | --- | --- | --- | --- | --- | --- | --- | --- | --- | --- | --- | --- |
| **Age (ref: ≤30)** | | | | | | | | | | | |  |
| 31-40 | 1.08 | (0.88, 1.32) | 0.46 | 1.35 | | (0.95, 1.93) | 0.09 | 0.90 | | (0.56, 1.46) | 0.66 |  |
| 41-50 | 1.59 | (1.18, 2.15) | **<0.01** | 1.09 | | (0.87, 1.37) | 0.43 | 0.98 | | (0.66, 1.46) | 0.93 |  |
| 51-60 | 1.81 | (1.45, 2.25) | **<0.01** | 1.46 | | (0.99, 2.13) | **0.05** | 1.10 | | (0.71, 1.69) | 0.66 |  |
| ≥61 | 2.00 | (1.56, 2.57) | **<0.01** | 1.53 | | (0.93, 2.52) | 0.09 | 1.15 | | (0.73, 1.79) | 0.53 |  |
| **Sex (ref: Female)** | | | | | | | | | | | |  |
| Male | 1.13 | (0.99, 1.31) | 0.08 | 1.13 | | (0.70, 1.84) | 0.59 | 1.23 | | (0.75, 2.03) | 0.39 |  |
| **Ethnicity (ref: White)** | | | | | | | | | | | |  |
| Black | 1.53 | (1.20, 1.94) | **<0.01** | 1.02 | | (0.57, 1.83) | 0.95 | 1.30 | | (0.72, 2.34) | 0.35 |  |
| Asian | 0.90 | (0.58, 1.39) | 0.61 | 1.19 | | (0.69, 2.05) | 0.52 | 1.30 | | (0.58, 2.92) | 0.50 |  |
| Mixed | 0.82 | (0.55, 1.24) | 0.33 | 2.22 | | (0.96, 5.11) | 0.06 | 0.94 | | (0.44, 2.01) | 0.86 |  |
| Other | 0.90 | (0.42, 1.94) | 0.78 | 1.34 | | (0.69, 2.63) | 0.37 | 0.57 | | (0.08, 4.21) | 0.56 |  |
| **Relationship (ref: Single)** | | | | | | | | | | | |  |
| In a relationship | 1.28 | (1.04, 1.57) | **0.02** | 0.95 | | (0.77, 1.17) | 0.61 | 1.06 | | (0.79, 1.42) | 0.69 |  |
| **Job role (ref: Non-clinical)** | | | | | | | | | | | |  |
| Doctor | 0.96 | (0.50, 1.83) | 0.89 | Insufficient sample size | | | | | | | |  |
| Nurse | 0.97 | (0.75, 1.25) | 0.80 | 0.81 | | (0.57, 1.17) | 0.24 | 1.26 | | (1.04, 1.53) | **0.02** |  |
| Other clinical | 0.95 | (0.75, 1.19) | 0.62 | 0.93 | | (0.81, 1.06) | 0.24 | 1.17 | | (0.95, 1.45) | 0.12 |  |
| **Pay grade (ref: AfC 5 and below)** | | | | | | | | | | | |  |
| AfC 6 and above | 1.37 | (1.02, 1.83) | **0.04** | 0.80 | | (0.57, 1.13) | 0.19 | 0.78 | | (0.61, 1.00) | **0.05** |  |
| **Setting (ref: Other hospital)** | | | | | | | | | | | |  |
| Accident & Emergency | 1.41 | (0.72, 2.78) | 0.29 | 1.03 | | (0.26, 4.07) | 0.97 | 2.02 | | (0.33, 12.25) | 0.42 |  |
| ICU/Critical Care | 0.85 | (0.61, 1.19) | 0.32 | 0.86 | | (0.49, 1.52) | 0.59 | 0.89 | | (0.43, 1.84) | 0.74 |  |
| Community | 0.95 | (0.79, 1.13) | 0.52 | 1.00 | | (0.67, 1.50) | 1.00 | 0.87 | | (0.69, 1.09) | 0.20 |  |
| Non-patient-facing | 1.25 | (0.94, 1.67) | 0.12 | 1.81 | | (0.97, 3.38) | 0.06 | 1.18 | | (0.75, 1.85) | 0.45 |  |
| **PPE (ref: Never/ Sometimes)** | | | | | | | | | | | |  |
| Often/ Always | 0.96 | (0.78, 1.18) | 0.65 | 1.28 | | (0.65, 2.52) | 0.45 | 1.72 | | (1.09, 2.72) | 0.02 |  |
| **Colleague support (ref: Not at all/A little)** | | | | | | | | | | | |  |
| Moderately/ Extremely | 1.00 | (0.68, 1.48) | 0.99 | 0.90 | | (0.60, 1.35) | 0.60 | 0.96 | | (0.71, 1.31) | 0.80 |  |
| **Manager support (ref: Not at all/A little)** | | | | | | | | | | | |  |
| Moderately/ Extremely | 1.29 | (0.98, 1.70) | 0.07 | 0.97 | | (0.71, 1.32) | 0.82 | 1.12 | | (0.87, 1.45) | 0.36 |  |
| **Moral injury (ref: below cut-off)** | | | | | | | | | | | |  |
| Met cut-off | 0.78 | (0.65, 0.92) | **0.01** | 1.18 | | (0.97, 1.44) | 0.09 | 0.94 | | (0.61, 1.44) | 0.77 |  |
| **Burden period (ref: Time 2 – low pressure)** | | | | | | | | | | | |  |
| T1 | 1.01 | (0.87, 1.17) | 0.92 | 0.99 | | (0.90, 1.09) | 0.86 | 0.81 | | (0.68, 0.96) | 0.02 |  |
| T3 | 0.82 | (0.71, 0.94) | **0.01** | 0.99 | | (0.50, 1.94) | 0.96 | 0.98 | | (0.76, 1.26) | 0.87 |  |
| **BRS (ref: below cut-off)** | | | | | | | | | | | |  |
| Met BRS baseline cut-off |  |  |  | 0.80 | | (0.57, 1.12) | 0.17 | 0.71 | | (0.57, 0.87) | **<0.01** |  |

Table 12 PTGI regression analyses

|  | **6 months**  AOR (95%CI) p≤ | | | **12 months**  AOR (95%CI) p≤ | | | **32 months**  AOR (95%CI) p≤ | | |
| --- | --- | --- | --- | --- | --- | --- | --- | --- | --- |
| **Age (ref: ≤30)** | | | | | | | | | |
| 31-40 | 1.05 | (0.55, 2.01) | 0.88 | 0.74 | (0.39, 1.39) | 0.33 | 0.68 | (0.39, 1.20) | 0.17 |
| 41-50 | 0.95 | (0.54, 1.65) | 0.84 | 0.63 | (0.41, 0.95) | 0.03 | 0.56 | (0.30, 1.01) | 0.06 |
| 51-60 | 0.92 | (0.52, 1.61) | 0.75 | 0.80 | (0.49, 1.33) | 0.37 | 0.66 | (0.32, 1.38) | 0.25 |
| ≥61 | 0.85 | (0.43, 1.69) | 0.62 | 1.42 | (0.87, 2.33) | 0.15 | 0.79 | (0.37, 1.67) | 0.51 |
| **Sex (ref: Female)** | | | | | | | | | |
| Male | 0.61 | (0.44, 0.85) | **0.01** | 0.63 | (0.40, 1.00) | **0.05** | 0.88 | (0.60, 1.29) | 0.48 |
| **Ethnicity (ref: White)** | | | | | | | | | |
| Black | 1.50 | (0.91, 2.46) | 0.10 | 2.76 | (1.70, 4.47) | **<0.01** | 2.11 | (0.68, 6.59) | 0.18 |
| Asian | 3.07 | (2.27, 4.15) | **<0.01** | 3.40 | (1.77, 6.55) | **<0.01** | 1.61 | (0.77, 3.38) | 0.19 |
| Mixed | 1.93 | (0.89, 4.21) | 0.09 | 2.04 | (0.82, 5.11) | 0.12 | 2.38 | (0.69, 8.21) | 0.16 |
| Other | 5.63 | (3.18, 9.95) | **<0.01** | 1.20 | (0.51, 2.83) | 0.66 | 6.91 | (3.02, 15.77) | **<0.01** |
| **Relationship (ref: Single)** | | | | | | | | | |
| In a relationship | 1.06 | (0.90, 1.25) | 0.47 | 1.34 | (1.06, 1.70) | **0.02** | 1.15 | (0.89, 1.49) | 0.26 |
| **Job role (ref: Non-clinical)** | | | | | | | | | |
| Doctor | 1.40 | (0.72, 2.72) | 0.29 | 0.37 | (0.03, 4.61) | 0.42 | 3.00 | (1.48, 6.06) | **<0.01** |
| Nurse | 1.23 | (0.97, 1.57) | 0.08 | 1.12 | (0.80, 1.58) | 0.48 | 1.66 | (1.13, 2.44) | **0.01** |
| Other clinical | 1.02 | (0.85, 1.23) | 0.80 | 1.06 | (0.72, 1.54) | 0.76 | 1.25 | (0.73, 2.14) | 0.39 |
| **Pay grade (ref: AfC 5 and below)** | | | | | | | | | |
| AfC 6 and above | 0.81 | (0.63, 1.03) | 0.08 | 0.95 | (0.69, 1.30) | 0.73 | 0.79 | (0.62, 1.00) | **0.05** |
| **Setting (ref: Other hospital)** | | | | | | | | | |
| Accident & Emergency | 0.66 | (0.31, 1.39) | 0.25 | 0.75 | (0.33, 1.69) | 0.46 | 2.13 | (0.70, 6.44) | 0.17 |
| ICU/Critical Care | 1.22 | (0.77, 1.93) | 0.38 | 0.82 | (0.34, 1.96) | 0.64 | 1.15 | (0.51, 2.56) | 0.72 |
| Community | 1.33 | (1.05, 1.68) | **0.02** | 1.33 | (1.04, 1.69) | **0.03** | 1.53 | (0.85, 2.76) | 0.14 |
| Non-patient-facing | 1.42 | (0.87, 2.33) | 0.15 | 1.70 | (0.88, 3.29) | 0.11 | 1.51 | (0.78, 2.93) | 0.20 |
| **PPE (ref: Never/ Sometimes)** | | | | | | | | | |
| Often/ Always | 1.02 | (0.78, 1.33) | 0.90 | 0.92 | (0.56, 1.50) | 0.72 | 1.03 | (0.71, 1.49) | 0.88 |
| **Colleague support (ref: Not at all/A little)** | | | | | | | | | |
| Moderately/Extremely | 1.19 | (0.79, 1.79) | 0.38 | 0.58 | (0.30, 1.12) | 0.10 | 0.57 | (0.30, 1.09) | 0.08 |
| **Manager support (ref: Not at all/A little)** | | | | | | | | | |
| Moderately/Extremely | 1.42 | (1.02, 1.98) | **0.04** | 1.14 | (0.69, 1.90) | 0.59 | 1.43 | (0.96, 2.14) | 0.08 |
| **Moral injury (ref: below cut-off)** | | | | | | | | | |
| Met cut-off | 1.33 | (1.06, 1.66) | **0.02** | 1.18 | (0.93, 1.50) | 0.16 | 0.99 | (0.73, 1.35) | 0.95 |
| **Burden period (ref: Time 2 – low pressure)** | | | | | | | | | |
| T1 | 1.03 | (0.70, 1.50) | 0.88 | 1.14 | (0.70, 1.86) | 0.57 | 1.41 | (0.99, 2.00) | 0.06 |
| T3 | 0.98 | (0.78, 1.23) | 0.85 | 0.98 | (0.65, 1.48) | 0.93 | 1.12 | (0.83, 1.51) | 0.45 |
| **BRS (ref: below cut-off)** | | | | | | | | | |
| Met BRS 6 months cut-off | - | - | - | 7.30 | (5.28, 10.08) | **<0.01** | 2.96 | (1.87, 4.68) | **<0.01** |
