# Supplementary material for: Mental health of healthcare workers in England during the first three years of the COVID-19 pandemic: The NHS CHECK study cohort
Source: PLoS One. 2026 Jun 26;21(6):e0350918. doi: 10.1371/journal.pone.0350918 (PMC13309052; doi:10.1371/journal.pone.0350918)
Supplement: Attachment — Submitted filename: Response to reviewers comments.docx [file pone.0350918.s003.docx]

| **Reviewer comment** | **Response** |
| --- | --- |
| **Reviewer #1** | |
| (1) The study title is okay. | We thank the reviewer for their helpful comments. |
| (2) The abstract needs minor revision. Although the abstract reads densely and places little emphasis on theoretical foundation or novelty, it is clear, thorough, and methodologically sound. The findings are succinctly summarized, but the implications—which should highlight particular practical approaches and possible advances in occupational mental health research—could be more critically stated. | While the paper did not aim to be theoretically focussed, we believe we have outlined the novelty of the large and weighted sample in the abstract. We would be happy to add further details if the editor is willing to extend the word limit for the abstract. |
| (3) The background also needs minor revision. It offers a thorough and organized summary of the body of research on the effects of COVID-19 on healthcare workers' mental health. It persuasively argues for the importance of the NHS CHECK study and draws attention to research gaps, especially the disregard for non-clinical staff. It could, however, more effectively synthesize findings to create a stronger conceptual justification because it is a little descriptive. The explanation of earlier discrepancies (e.g. G. between HCW and overall population distress) requires more precise incorporation into the objectives of the study. To improve focus and cut down on repetition, some parts could be simplified. Please include cross-cultural research on the mental health outcomes of the COVID-19 pandemic and add the following research in the background: Tiwari, G. K., Singh, A. K., Parihar, P., Pandey, R., Sharma, D. N., & Rai, P. K. (2023). Understanding the perceived psychological distress and health outcomes of children during COVID-19 pandemic. Educational and Developmental Psychologist, 40(1), 103–114. <https://doi.org/10.1080/20590776.2021.1899749> Tiwari, G. K., Tiwari, R. P., Pandey, R., Ray, B., Dwivedi, A., Sharma, D. N., Singh, P., Tiwari, A. K., & Singh, A. K. (2024). Perceived Life Outcomes of Indian Children During the Early Phase of the COVID-19 Lockdown: The Protective Roles of Joint and Nuclear Families. Journal of Research and Health, 14(1), 43–54. <https://doi.org/10.32598/JRH.14.1.1992.4> | We thank the reviewer for their kind comments. We are somewhat confused, however, by the suggested additional references, as both seem to be about children, whereas the sample in this study were working age adults. As the Background section is already quite short, we believe cutting it further would detract from our ability to outline the problem and need to the study. We are not clear which parts of the Background section the reviewer feels are repetitive, and haven’t identified any ourselves, but would be happy to edit this section if the editor can share which parts they would like cut. |
| (4) The methods section also needs improvements. Although the study uses a strong longitudinal cohort design with suitable follow-up intervals, there are a number of methodological issues. It's possible that selection bias was introduced during participant recruitment through internal communication channels, reducing representativeness. Over time, attrition bias and response accuracy become issues when self-reported online data is used. Despite the use of validated measures, data uniformity was diminished by varying survey lengths. Although the imputation and weighting techniques are suitable, they are not sufficiently supported. Response rates, how to handle missing data, and steps to guarantee validity and reliability across time-points all require more clarification. | We already acknowledge the possibility of selection bias (lines 384-385, and 388-389) in the ‘Strengths and limitations’ section, but would be happy to move this to the Methods section if requested by the editor.  We not believe the slightly different survey lengths between waves of data collection is a limitation. We have made clear that the surveys at each time point included most of the same measures each time, but were not identical (to account for changing research and policy priorities) (lines 132-134).  We are not clear what the reviewer means by the imputation and weighting techniques being suitable but not being ‘sufficiently supported’. We would be happy to address this if further clarity can be provided.  We believe we have already clearly outlined the response rate, how we handled missing data, and how we guaranteed validity and reliability, but would be happy to add to this if the reviewer can explain what additional information they are requesting. |
| (5) The results section also needs revision. The results section demonstrates thorough handling of extensive longitudinal data and is methodologically transparent and detailed. However, readability is hampered by too much tabular data, and certain tables (e.g. G. are not effectively incorporated into the story (Table 2). Significant attrition rates across time-points cast doubt on the representativeness and possible bias of longitudinal results. Weighted regression and VIF are suitable statistical controls, but interpretations are unclear regarding their applicability. There is little inferential commentary that connects findings to theoretical or contextual implications, and the description of outcomes (CMDs, PTSD, burnout) is primarily descriptive. | We appreciate the reviewer’s perspective, however we believe that providing full data in a table, with a summary of key points in the text is the way most readers will prefer the information to be presented. We would be happy to e.g. move Table 2 to an appendix if the editor believe this is necessary.  We have noted already in the Strengths and limitations section that there was attrition over time.  We are not sure what the reviewer means by ‘interpretations are unclear regarding their applicability’.  We agree with the reviewer that the description of outcomes is primarily descriptive, and this was indeed the aim of the paper. |
| (6) The discussion section also needs revision. It is thorough, organized, and skillfully incorporates the results with previously published works. It provides little theoretical interpretation, though, and is more descriptive than analytical. A few associations (e.g. G. ethnicity, gender, and so forth) are reported without sufficient investigation of the underlying mechanisms. A more thorough examination of the causal pathways, implications of the intervention, and contextual interpretation of the results would enhance this section. The limitations are discussed in a defensive manner, even though strengths and limitations are duly acknowledged. The impact and coherence of the discussion would be improved by placing more focus on the real-world applications and policy translation. Please include the following research from other cultural contexts to interpret the study findings: Tiwari, G. K., Rai, P. K., Dwivedi, A., Ray, B., Pandey, A., & Pandey, R. (2023). A Narrative Thematic Analysis of the perceived psychological distress and health outcomes in Indian adults during the early phase of the COVID-19 pandemic. Psychology: The Journal of the Hellenic Psychological Society, 28(1), 213–229. <https://doi.org/10.12681/psy_hps.28062> | We appreciate the reviewer’s kind words. While we agree that research is often usefully grounded in theory, as a descriptive paper that aimed to describe prevalence, we believe we should be wary of overinterpreting the results, or of assuming causality – the analyses are not appropriate to do so.  There was no intervention, so we are not sure what is meant by ‘implications of the intervention’.  We would be happy to amend the limitations section if requested by the editor, but believe it is helpful to outline both limitations, and what was done in attempts to mitigate them.  We believe that we have addressed real-world applications and policy implications in lines 394-405.  As the suggested paper does not focus on healthcare workers we believe it would be inappropriate to include this. |
| (7) The references are okay. Please correct it by following the journal’s guidelines. The tables and figures should be prepared following standard guidelines. | We believe the reference, tables, and figures are formatted according to the journal’s guidelines, but are happy to make any changes requested by the editor. |
| **Reviewer #2** |  |
| 1. Conceptual novelty is limited While the sample size is impressive, the theoretical contribution is limited. The introduction could benefit from integrating: occupational stress models, moral injury frameworks, and burnout theory. The paper is largely descriptive and confirms patterns already widely reported in COVID-19 mental health research. The discussion should better clarify what new knowledge this cohort provides beyond existing studies. | As above, while we agree that theory is important, we are wary of adding large quantities of additional text that goes beyond the remit of the paper. The paper is indeed descriptive: that was the primary aim. We have added in the Strengths section, “These strengths provide, to our knowledge, the best quality data available regarding HCW mental health and wellbeing.”. |
| 2. Cross-sectional analysis of a longitudinal cohort Although the study is designed as longitudinal, the current analysis treats the data cross-sectionally at each timepoint. This reduces the potential contribution of the cohort. Include at least one longitudinal model if possible (e.g., mixed effects or growth curve) to justify the cohort design. | We have carried out trajectory modelling in a separate paper, and have noted this in lines 221-223, “This paper presents descriptive data at multiple time points, and we provide longitudinal trajectory modelling in a separate paper.”. The trajectory modelling paper has been accepted for publication, and we anticipate we will be able to provide a full reference to be added here before this paper is published. |
| 3. Response bias and representativeness The baseline response rate is only 16%, which raises serious concerns regarding: • self-selection bias • overrepresentation of distressed participants Although weighting was applied, the manuscript should discuss this limitation more explicitly. | We believe we have discussed this limitation explicitly, in lines 390-392. However, we have added additional detail noting the point about overrepresentation of distressed participants, “Although our 16% baseline response rate exceeded similar studies, it was suboptimal, and may have either overrepresented or underrepresented distressed participants. On balance, given that those most distressed are likely to exit the workforce either temporarily or permanently, it seems more likely that results underrepresent than overrepresent prevalence.” (lines 390-393). |
| 4. Attrition across timepoints Participant numbers decrease substantially: Baseline: 22,092 6 months: 10,514 12 months: 11,998 (includes replenishment cohort) 32 months: 6,991 Attrition could bias the results if individuals with worse mental health dropped out. The authors should provide: • attrition analysis • comparison of responders vs non-responders. | We agree that providing attrition analyses comparing responders vs non-responders might be of interest to some readers. We have added these analyses to the Supplementary files, and added a paragraph in the Results section: “We assessed differences between responders and non-responders by key demographics (age, sex, ethnicity, job role) at six, 12, and 32 months, using T tests for continuous variables and Chi2 for categorical variables. At all three time points, responders (vs non-responders) were likely to be older by around 3-5 years (p<0.001). At six months, responders were more likely to be women (46% vs 44%, p=0.022), though there were no statistically significant differences in responders by sex at 12 or 32 months. At all three time points, responders were more likely to be White, and in the non-clinical staff group, and though statistically significant, differences were small. Full details can be found in Supplementary file 2.” (lines 244-251). We have also added additional detail to the ‘Strengths and limitations’ section noting this (lines 398-401). |
| 5. Interpretation of CMD prevalence The authors report that ~50% of staff met GHQ-12 caseness, which is extremely high. However: GHQ-12 “caseness” indicates psychological distress, not clinical disorder. The manuscript occasionally implies clinical diagnosis. Clarify throughout that this represents probable distress rather than confirmed mental disorder. | We agree that this is an important distinction, and we believe that we have been clear about this throughout. If there are specific instances where the reviewer can point to implications of clinical diagnosis we would be happy to amend these. |
| 6. Moral injury measurement limitations The study dichotomizes the Moral Injury Events Scale (MIES) using endorsement of a single item. More nuanced analysis (e.g., subscales) would strengthen the findings. | We agree that more nuanced analyses might be interesting to some readers, but in using a dichotomised version of the MIES we attempted to balance inclusion of an interesting outcome measure with what could feasibly be included in a broad paper. We have provided analyses focussing specifically on moral injury in separate papers. |
| Typographical errors: Example: “writeen consent” instead of written consent. Line 421 contains: “Error! Not a valid bookmark self-reference”. This must be corrected before publication. | We cannot find any instance of the typo, ‘writeen’.  Similarly, we cannot find any ‘Error’. We are happy to correct any typos that the editor is aware of. |
| Data availability statement: The manuscript states that data are available upon request, which does not fully comply with PLOS ONE data sharing policy. Authors should deposit data in a repository or justify restrictions. | We received ethical approvals to share data if requested, but not to deposit the full data in a repository. This is already stated clearly in the ‘Availability of data and materials’ section (lines 649-652). |
