# Supplementary material for: Mental health of healthcare workers in England during the first three years of the COVID-19 pandemic: The NHS CHECK study cohort
Source: PLoS One. 2026 Jun 26;21(6):e0350918. doi: 10.1371/journal.pone.0350918 (PMC13309052; doi:10.1371/journal.pone.0350918)
Supplement: Peer review history

# Peer review: Decision Letter 0

**DOI:** 10.1371/journal.pone.0350918.r001

**Authors:** Giuseppe Marano

19 Mar 2026

PONE-D-25-40092

Mental health of healthcare workers in England during the first three years of the COVID-19 pandemic: the NHS CHECK study cohort

PLOS One

Dear Dr. Lamb,

Thank you for submitting your manuscript to PLOS ONE. After careful consideration, we feel that it has merit but does not fully meet PLOS ONE’s publication criteria as it currently stands. Therefore, we invite you to submit a revised version of the manuscript that addresses the points raised during the review process.

I strongly encourage you to carefully address all the reviewers’ comments, ensuring that each point is thoroughly considered and adequately incorporated into the revised manuscript. Particular attention should be given to the Discussion section, which requires substantial improvement and deeper development. A more rigorous examination of causal pathways, the implications of the intervention and a well-contextualized interpretation of the results would significantly strengthen this section. The limitations should be discussed in a balanced and critical way, clearly acknowledging both strengths and weaknesses.

Please submit your revised manuscript by May 03 2026 11:59PM. If you will need more time than this to complete your revisions, please reply to this message or contact the journal office at plosone@plos.org. When you're ready to submit your revision, log on to https://www.editorialmanager.com/pone/ and select the 'Submissions Needing Revision' folder to locate your manuscript file.

If you would like to make changes to your financial disclosure, please include your updated statement in your cover letter. Guidelines for resubmitting your figure files are available below the reviewer comments at the end of this letter.

If applicable, we recommend that you deposit your laboratory protocols in protocols.io to enhance the reproducibility of your results. Protocols.io assigns your protocol its own identifier (DOI) so that it can be cited independently in the future. For instructions see: https://journals.plos.org/plosone/s/submission-guidelines#loc-laboratory-protocols. Additionally, PLOS ONE offers an option for publishing peer-reviewed Lab Protocol articles, which describe protocols hosted on protocols.io. Read more information on sharing protocols at https://plos.org/protocols?utm_medium=editorial-email&utm_source=authorletters&utm_campaign=protocols.

We look forward to receiving your revised manuscript.

Kind regards,

Giuseppe Marano

Academic Editor

PLOS One

Journal Requirements:

When submitting your revision, we need you to address these additional requirements.

1. Please ensure that your manuscript meets PLOS ONE's style requirements, including those for file naming. The PLOS ONE style templates can be found at

https://journals.plos.org/plosone/s/file?id=wjVg/PLOSOne_formatting_sample_main_body.pdf and

https://journals.plos.org/plosone/s/file?id=ba62/PLOSOne_formatting_sample_title_authors_affiliations.pdf

2. Thank you for stating the following financial disclosure: [NHS CHECK received funding from the Medical Research Council (MR/V034405/1), UCL/Wellcome (ISSF3/H17RCO/C3), Rosetrees (M952), Economic and Social Research Council (ES/V009931/1),].

Please state what role the funders took in the study.  If the funders had no role, please state: ""The funders had no role in study design, data collection and analysis, decision to publish, or preparation of the manuscript.""

If this statement is not correct you must amend it as needed.

Please include this amended Role of Funder statement in your cover letter; we will change the online submission form on your behalf.

3. We note that the grant information you provided in the ‘Funding Information’ and ‘Financial Disclosure’ sections do not match.

When you resubmit, please ensure that you provide the correct grant numbers for the awards you received for your study in the ‘Funding Information’ section.

4. Thank you for stating the following in the Competing Interests section: [I have read the journal's policy and the authors of this manuscript have the following competing interests:

SW has received speaker fees from Swiss Re for two webinars on the epidemiological impact of the COVID 19 pandemic on mental health.

NG reports a potential COI with NHSEI,  during the conduct of the study. He is the managing director of March on Stress Ltd which has provided training for a number of NHS organisations.

Other authors report no competing interests.].

Please confirm that this does not alter your adherence to all PLOS ONE policies on sharing data and materials, by including the following statement: ""This does not alter our adherence to  PLOS ONE policies on sharing data and materials.” (as detailed online in our guide for authors http://journals.plos.org/plosone/s/competing-interests).  If there are restrictions on sharing of data and/or materials, please state these. Please note that we cannot proceed with consideration of your article until this information has been declared.

Please include your updated Competing Interests statement in your cover letter; we will change the online submission form on your behalf.

5. In the online submission form, you indicated that [The datasets generated and/or analysed during the current study are not publicly available due to lack of ethical approval for full datasets to be shared publicly, but are available from the corresponding author on reasonable request.].

All PLOS journals now require all data underlying the findings described in their manuscript to be freely available to other researchers, either 1. In a public repository, 2. Within the manuscript itself, or 3. Uploaded as supplementary information.

This policy applies to all data except where public deposition would breach compliance with the protocol approved by your research ethics board. If your data cannot be made publicly available for ethical or legal reasons (e.g., public availability would compromise patient privacy), please explain your reasons on resubmission and your exemption request will be escalated for approval.

6. Please include captions for your Supporting Information files at the end of your manuscript, and update any in-text citations to match accordingly. Please see our Supporting Information guidelines for more information: http://journals.plos.org/plosone/s/supporting-information.

7. If the reviewer comments include a recommendation to cite specific previously published works, please review and evaluate these publications to determine whether they are relevant and should be cited. There is no requirement to cite these works unless the editor has indicated otherwise.

[Note: HTML markup is below. Please do not edit.]

Reviewers' comments:

Reviewer's Responses to Questions

**Comments to the Author**

1. Is the manuscript technically sound, and do the data support the conclusions?

The manuscript must describe a technically sound piece of scientific research with data that supports the conclusions. Experiments must have been conducted rigorously, with appropriate controls, replication, and sample sizes. The conclusions must be drawn appropriately based on the data presented.

Reviewer #1: Yes

Reviewer #2: Yes

**********

2. Has the statistical analysis been performed appropriately and rigorously?

Reviewer #1: Yes

Reviewer #2: Yes

**********

3. Have the authors made all data underlying the findings in their manuscript fully available?

The PLOS Data policy requires authors to make all data underlying the findings described in their manuscript fully available without restriction, with rare exception (please refer to the Data Availability Statement in the manuscript PDF file). The data should be provided as part of the manuscript or its supporting information, or deposited to a public repository. For example, in addition to summary statistics, the data points behind means, medians and variance measures should be available. If there are restrictions on publicly sharing data—e.g. participant privacy or use of data from a third party—those must be specified.

Reviewer #1: Yes

Reviewer #2: No

**********

4. Is the manuscript presented in an intelligible fashion and written in standard English?

PLOS ONE does not copyedit accepted manuscripts, so the language in submitted articles must be clear, correct, and unambiguous. Any typographical or grammatical errors should be corrected at revision, so please note any specific errors here.

Reviewer #1: Yes

Reviewer #2: Yes

**********

5. Review Comments to the Author

Please use the space provided to explain your answers to the questions above. You may also include additional comments for the author, including concerns about dual publication, research ethics, or publication ethics. (Please upload your review as an attachment if it exceeds 20,000 characters)

Reviewer #1: The study's goals were to identify the staff members most at risk of negative mental health outcomes and investigate differences in mental health over a three-year period across all staff types. My observations are given below:

(1) The study title is okay.

(2) The abstract needs minor revision. Although the abstract reads densely and places little emphasis on theoretical foundation or novelty, it is clear, thorough, and methodologically sound. The findings are succinctly summarized, but the implications—which should highlight particular practical approaches and possible advances in occupational mental health research—could be more critically stated.

(3) The background also needs minor revision. It offers a thorough and organized summary of the body of research on the effects of COVID-19 on healthcare workers' mental health. It persuasively argues for the importance of the NHS CHECK study and draws attention to research gaps, especially the disregard for non-clinical staff. It could, however, more effectively synthesize findings to create a stronger conceptual justification because it is a little descriptive. The explanation of earlier discrepancies (e.g. G. between HCW and overall population distress) requires more precise incorporation into the objectives of the study. To improve focus and cut down on repetition, some parts could be simplified. Please include cross-cultural research on the mental health outcomes of the COVID-19 pandemic and add the following research in the background:

Tiwari, G. K., Singh, A. K., Parihar, P., Pandey, R., Sharma, D. N., & Rai, P. K. (2023). Understanding the perceived psychological distress and health outcomes of children during COVID-19 pandemic. Educational and Developmental Psychologist, 40(1), 103–114. https://doi.org/10.1080/20590776.2021.1899749

Tiwari, G. K., Tiwari, R. P., Pandey, R., Ray, B., Dwivedi, A., Sharma, D. N., Singh, P., Tiwari, A. K., & Singh, A. K. (2024). Perceived Life Outcomes of Indian Children During the Early Phase of the COVID-19 Lockdown: The Protective Roles of Joint and Nuclear Families. Journal of Research and Health, 14(1), 43–54. https://doi.org/10.32598/JRH.14.1.1992.4

(4) The methods section also needs improvements. Although the study uses a strong longitudinal cohort design with suitable follow-up intervals, there are a number of methodological issues. It's possible that selection bias was introduced during participant recruitment through internal communication channels, reducing representativeness. Over time, attrition bias and response accuracy become issues when self-reported online data is used. Despite the use of validated measures, data uniformity was diminished by varying survey lengths. Although the imputation and weighting techniques are suitable, they are not sufficiently supported. Response rates, how to handle missing data, and steps to guarantee validity and reliability across time-points all require more clarification.

(5) The results section also needs revision. The results section demonstrates thorough handling of extensive longitudinal data and is methodologically transparent and detailed. However, readability is hampered by too much tabular data, and certain tables (e.g. G. are not effectively incorporated into the story (Table 2). Significant attrition rates across time-points cast doubt on the representativeness and possible bias of longitudinal results. Weighted regression and VIF are suitable statistical controls, but interpretations are unclear regarding their applicability. There is little inferential commentary that connects findings to theoretical or contextual implications, and the description of outcomes (CMDs, PTSD, burnout) is primarily descriptive.

(6) The discussion section also needs revision. It is thorough, organized, and skillfully incorporates the results with previously published works. It provides little theoretical interpretation, though, and is more descriptive than analytical. A few associations (e.g. G. ethnicity, gender, and so forth) are reported without sufficient investigation of the underlying mechanisms. A more thorough examination of the causal pathways, implications of the intervention, and contextual interpretation of the results would enhance this section. The limitations are discussed in a defensive manner, even though strengths and limitations are duly acknowledged. The impact and coherence of the discussion would be improved by placing more focus on the real-world applications and policy translation. Please include the following research from other cultural contexts to interpret the study findings:

Tiwari, G. K., Rai, P. K., Dwivedi, A., Ray, B., Pandey, A., & Pandey, R. (2023). A Narrative Thematic Analysis of the perceived psychological distress and health outcomes in Indian adults during the early phase of the COVID-19 pandemic. Psychology: The Journal of the Hellenic Psychological Society, 28(1), 213–229. https://doi.org/10.12681/psy_hps.28062

(7) The references are okay. Please correct it by following the journal’s guidelines. The tables and figures should be prepared following standard guidelines.

Reviewer #2: Comments for authors

1. Conceptual novelty is limited

While the sample size is impressive, the theoretical contribution is limited. The introduction could benefit from integrating: occupational stress models, moral injury frameworks, and burnout theory.

The paper is largely descriptive and confirms patterns already widely reported in COVID-19 mental health research. The discussion should better clarify what new knowledge this cohort provides beyond existing studies.

2. Cross-sectional analysis of a longitudinal cohort

Although the study is designed as longitudinal, the current analysis treats the data cross-sectionally at each timepoint.

This reduces the potential contribution of the cohort. Include at least one longitudinal model if possible (e.g., mixed effects or growth curve) to justify the cohort design.

3. Response bias and representativeness

The baseline response rate is only 16%, which raises serious concerns regarding:

• self-selection bias

• overrepresentation of distressed participants

Although weighting was applied, the manuscript should discuss this limitation more explicitly.

4. Attrition across timepoints

Participant numbers decrease substantially:

Baseline: 22,092

6 months: 10,514

12 months: 11,998 (includes replenishment cohort)

32 months: 6,991

Attrition could bias the results if individuals with worse mental health dropped out.

The authors should provide:

• attrition analysis

• comparison of responders vs non-responders.

5. Interpretation of CMD prevalence

The authors report that ~50% of staff met GHQ-12 caseness, which is extremely high.

However:

GHQ-12 “caseness” indicates psychological distress, not clinical disorder. The manuscript occasionally implies clinical diagnosis. Clarify throughout that this represents probable distress rather than confirmed mental disorder.

6. Moral injury measurement limitations

The study dichotomizes the Moral Injury Events Scale (MIES) using endorsement of a single item. More nuanced analysis (e.g., subscales) would strengthen the findings.

Also:

Typographical errors: Example: “writeen consent” instead of written consent.

Line 421 contains: “Error! Not a valid bookmark self-reference”. This must be corrected before publication.

Data availability statement: The manuscript states that data are available upon request, which does not fully comply with PLOS ONE data sharing policy. Authors should deposit data in a repository or justify restrictions.

**********

6. PLOS authors have the option to publish the peer review history of their article (what does this mean?). If published, this will include your full peer review and any attached files.

If you choose “no”, your identity will remain anonymous but your review may still be made public.

**Do you want your identity to be public for this peer review?**  For information about this choice, including consent withdrawal, please see our Privacy Policy.

Reviewer #1: **Yes:** Gyanesh Kumar Tiwari

Reviewer #2: No

**********

[NOTE: If reviewer comments were submitted as an attachment file, they will be attached to this email and accessible via the submission site. Please log into your account, locate the manuscript record, and check for the action link "View Attachments". If this link does not appear, there are no attachment files.]

To ensure your figures meet our technical requirements, please review our figure guidelines: https://journals.plos.org/plosone/s/figures

You may also use PLOS’s free figure tool, NAAS, to help you prepare publication quality figures: https://journals.plos.org/plosone/s/figures#loc-tools-for-figure-preparation.

NAAS will assess whether your figures meet our technical requirements by comparing each figure against our figure specifications.

# Author comment: Author response to Decision Letter 1

**DOI:** 10.1371/journal.pone.0350918.r002

10 Apr 2026

Reviewer comment Response

Reviewer #1

(1) The study title is okay. We thank the reviewer for their helpful comments.

(2) The abstract needs minor revision. Although the abstract reads densely and places little emphasis on theoretical foundation or novelty, it is clear, thorough, and methodologically sound. The findings are succinctly summarized, but the implications—which should highlight particular practical approaches and possible advances in occupational mental health research—could be more critically stated.

While the paper did not aim to be theoretically focussed, we believe we have outlined the novelty of the large and weighted sample in the abstract. We would be happy to add further details if the editor is willing to extend the word limit for the abstract.

(3) The background also needs minor revision. It offers a thorough and organized summary of the body of research on the effects of COVID-19 on healthcare workers' mental health. It persuasively argues for the importance of the NHS CHECK study and draws attention to research gaps, especially the disregard for non-clinical staff. It could, however, more effectively synthesize findings to create a stronger conceptual justification because it is a little descriptive. The explanation of earlier discrepancies (e.g. G. between HCW and overall population distress) requires more precise incorporation into the objectives of the study. To improve focus and cut down on repetition, some parts could be simplified. Please include cross-cultural research on the mental health outcomes of the COVID-19 pandemic and add the following research in the background:

Tiwari, G. K., Singh, A. K., Parihar, P., Pandey, R., Sharma, D. N., & Rai, P. K. (2023). Understanding the perceived psychological distress and health outcomes of children during COVID-19 pandemic. Educational and Developmental Psychologist, 40(1), 103–114. https://doi.org/10.1080/20590776.2021.1899749

Tiwari, G. K., Tiwari, R. P., Pandey, R., Ray, B., Dwivedi, A., Sharma, D. N., Singh, P., Tiwari, A. K., & Singh, A. K. (2024). Perceived Life Outcomes of Indian Children During the Early Phase of the COVID-19 Lockdown: The Protective Roles of Joint and Nuclear Families. Journal of Research and Health, 14(1), 43–54. https://doi.org/10.32598/JRH.14.1.1992.4

We thank the reviewer for their kind comments. We are somewhat confused, however, by the suggested additional references, as both seem to be about children, whereas the sample in this study were working age adults. As the Background section is already quite short, we believe cutting it further would detract from our ability to outline the problem and need to the study. We are not clear which parts of the Background section the reviewer feels are repetitive, and haven’t identified any ourselves, but would be happy to edit this section if the editor can share which parts they would like cut.

(4) The methods section also needs improvements. Although the study uses a strong longitudinal cohort design with suitable follow-up intervals, there are a number of methodological issues. It's possible that selection bias was introduced during participant recruitment through internal communication channels, reducing representativeness. Over time, attrition bias and response accuracy become issues when self-reported online data is used. Despite the use of validated measures, data uniformity was diminished by varying survey lengths. Although the imputation and weighting techniques are suitable, they are not sufficiently supported. Response rates, how to handle missing data, and steps to guarantee validity and reliability across time-points all require more clarification.

We already acknowledge the possibility of selection bias (lines 384-385, and 388-389) in the ‘Strengths and limitations’ section, but would be happy to move this to the Methods section if requested by the editor.

We not believe the slightly different survey lengths between waves of data collection is a limitation. We have made clear that the surveys at each time point included most of the same measures each time, but were not identical (to account for changing research and policy priorities) (lines 132-134).

We are not clear what the reviewer means by the imputation and weighting techniques being suitable but not being ‘sufficiently supported’. We would be happy to address this if further clarity can be provided.

We believe we have already clearly outlined the response rate, how we handled missing data, and how we guaranteed validity and reliability, but would be happy to add to this if the reviewer can explain what additional information they are requesting.

(5) The results section also needs revision. The results section demonstrates thorough handling of extensive longitudinal data and is methodologically transparent and detailed. However, readability is hampered by too much tabular data, and certain tables (e.g. G. are not effectively incorporated into the story (Table 2). Significant attrition rates across time-points cast doubt on the representativeness and possible bias of longitudinal results. Weighted regression and VIF are suitable statistical controls, but interpretations are unclear regarding their applicability. There is little inferential commentary that connects findings to theoretical or contextual implications, and the description of outcomes (CMDs, PTSD, burnout) is primarily descriptive.

We appreciate the reviewer’s perspective, however we believe that providing full data in a table, with a summary of key points in the text is the way most readers will prefer the information to be presented. We would be happy to e.g. move Table 2 to an appendix if the editor believe this is necessary.

We have noted already in the Strengths and limitations section that there was attrition over time.

We are not sure what the reviewer means by ‘interpretations are unclear regarding their applicability’.

We agree with the reviewer that the description of outcomes is primarily descriptive, and this was indeed the aim of the paper.

(6) The discussion section also needs revision. It is thorough, organized, and skillfully incorporates the results with previously published works. It provides little theoretical interpretation, though, and is more descriptive than analytical. A few associations (e.g. G. ethnicity, gender, and so forth) are reported without sufficient investigation of the underlying mechanisms. A more thorough examination of the causal pathways, implications of the intervention, and contextual interpretation of the results would enhance this section. The limitations are discussed in a defensive manner, even though strengths and limitations are duly acknowledged. The impact and coherence of the discussion would be improved by placing more focus on the real-world applications and policy translation. Please include the following research from other cultural contexts to interpret the study findings:

Tiwari, G. K., Rai, P. K., Dwivedi, A., Ray, B., Pandey, A., & Pandey, R. (2023). A Narrative Thematic Analysis of the perceived psychological distress and health outcomes in Indian adults during the early phase of the COVID-19 pandemic. Psychology: The Journal of the Hellenic Psychological Society, 28(1), 213–229. https://doi.org/10.12681/psy_hps.28062

We appreciate the reviewer’s kind words. While we agree that research is often usefully grounded in theory, as a descriptive paper that aimed to describe prevalence, we believe we should be wary of overinterpreting the results, or of assuming causality – the analyses are not appropriate to do so.

There was no intervention, so we are not sure what is meant by ‘implications of the intervention’.

We would be happy to amend the limitations section if requested by the editor, but believe it is helpful to outline both limitations, and what was done in attempts to mitigate them.

We believe that we have addressed real-world applications and policy implications in lines 394-405.

As the suggested paper does not focus on healthcare workers we believe it would be inappropriate to include this.

(7) The references are okay. Please correct it by following the journal’s guidelines. The tables and figures should be prepared following standard guidelines. We believe the reference, tables, and figures are formatted according to the journal’s guidelines, but are happy to make any changes requested by the editor.

Reviewer #2

1. Conceptual novelty is limited

While the sample size is impressive, the theoretical contribution is limited. The introduction could benefit from integrating: occupational stress models, moral injury frameworks, and burnout theory.

The paper is largely descriptive and confirms patterns already widely reported in COVID-19 mental health research. The discussion should better clarify what new knowledge this cohort provides beyond existing studies.

As above, while we agree that theory is important, we are wary of adding large quantities of additional text that goes beyond the remit of the paper. The paper is indeed descriptive: that was the primary aim. We have added in the Strengths section, “These strengths provide, to our knowledge, the best quality data available regarding HCW mental health and wellbeing.”.

2. Cross-sectional analysis of a longitudinal cohort

Although the study is designed as longitudinal, the current analysis treats the data cross-sectionally at each timepoint.

This reduces the potential contribution of the cohort. Include at least one longitudinal model if possible (e.g., mixed effects or growth curve) to justify the cohort design. We have carried out trajectory modelling in a separate paper, and have noted this in lines 221-223, “This paper presents descriptive data at multiple time points, and we provide longitudinal trajectory modelling in a separate paper.”. The trajectory modelling paper has been accepted for publication, and we anticipate we will be able to provide a full reference to be added here before this paper is published.

3. Response bias and representativeness

The baseline response rate is only 16%, which raises serious concerns regarding:

• self-selection bias

• overrepresentation of distressed participants

Although weighting was applied, the manuscript should discuss this limitation more explicitly. We believe we have discussed this limitation explicitly, in lines 390-392. However, we have added additional detail noting the point about overrepresentation of distressed participants, “Although our 16% baseline response rate exceeded similar studies, it was suboptimal, and may have either overrepresented or underrepresented distressed participants. On balance, given that those most distressed are likely to exit the workforce either temporarily or permanently, it seems more likely that results underrepresent than overrepresent prevalence.” (lines 390-393).

4. Attrition across timepoints

Participant numbers decrease substantially:

Baseline: 22,092

6 months: 10,514

12 months: 11,998 (includes replenishment cohort)

32 months: 6,991

Attrition could bias the results if individuals with worse mental health dropped out.

The authors should provide:

• attrition analysis

• comparison of responders vs non-responders. We agree that providing attrition analyses comparing responders vs non-responders might be of interest to some readers. We have added these analyses to the Supplementary files, and added a paragraph in the Results section: “We assessed differences between responders and non-responders by key demographics (age, sex, ethnicity, job role) at six, 12, and 32 months, using T tests for continuous variables and Chi2 for categorical variables. At all three time points, responders (vs non-responders) were likely to be older by around 3-5 years (p<0.001). At six months, responders were more likely to be women (46% vs 44%, p=0.022), though there were no statistically significant differences in responders by sex at 12 or 32 months. At all three time points, responders were more likely to be White, and in the non-clinical staff group, and though statistically significant, differences were small. Full details can be found in Supplementary file 2.” (lines 244-251). We have also added additional detail to the ‘Strengths and limitations’ section noting this (lines 398-401).

5. Interpretation of CMD prevalence

The authors report that ~50% of staff met GHQ-12 caseness, which is extremely high.

However:

GHQ-12 “caseness” indicates psychological distress, not clinical disorder. The manuscript occasionally implies clinical diagnosis. Clarify throughout that this represents probable distress rather than confirmed mental disorder. We agree that this is an important distinction, and we believe that we have been clear about this throughout. If there are specific instances where the reviewer can point to implications of clinical diagnosis we would be happy to amend these.

6. Moral injury measurement limitations

The study dichotomizes the Moral Injury Events Scale (MIES) using endorsement of a single item. More nuanced analysis (e.g., subscales) would strengthen the findings. We agree that more nuanced analyses might be interesting to some readers, but in using a dichotomised version of the MIES we attempted to balance inclusion of an interesting outcome measure with what could feasibly be included in a broad paper. We have provided analyses focussing specifically on moral injury in separate papers.

Typographical errors: Example: “writeen consent” instead of written consent.

Line 421 contains: “Error! Not a valid bookmark self-reference”. This must be corrected before publication.

We cannot find any instance of the typo, ‘writeen’.

Similarly, we cannot find any ‘Error’. We are happy to correct any typos that the editor is aware of.

Data availability statement: The manuscript states that data are available upon request, which does not fully comply with PLOS ONE data sharing policy. Authors should deposit data in a repository or justify restrictions. We received ethical approvals to share data if requested, but not to deposit the full data in a repository. This is already stated clearly in the ‘Availability of data and materials’ section (lines 649-652).

Attachment Submitted filename: Response to reviewers comments.docx

# Peer review: Decision Letter 1

**DOI:** 10.1371/journal.pone.0350918.r003

**Authors:** Giuseppe Marano

21 May 2026

Mental health of healthcare workers in England during the first three years of the COVID-19 pandemic: the NHS CHECK study cohort

PONE-D-25-40092R1

Dear Dr. Lamb,

We’re pleased to inform you that your manuscript has been judged scientifically suitable for publication and will be formally accepted for publication once it meets all outstanding technical requirements.

Within one week, you’ll receive an e-mail detailing the required amendments. When these have been addressed, you’ll receive a formal acceptance letter and your manuscript will be scheduled for publication.

An invoice will be generated when your article is formally accepted. Please note, if your institution has a publishing partnership with PLOS and your article meets the relevant criteria, all or part of your publication costs will be covered. Please make sure your user information is up-to-date by logging into Editorial Manager at Editorial Manager® and clicking the ‘Update My Information' link at the top of the page. For questions related to billing, please contact billing support.

If your institution or institutions have a press office, please notify them about your upcoming paper to help maximize its impact. If they’ll be preparing press materials, please inform our press team as soon as possible -- no later than 48 hours after receiving the formal acceptance. Your manuscript will remain under strict press embargo until 2 pm Eastern Time on the date of publication. For more information, please contact onepress@plos.org.

Kind regards,

Giuseppe Marano

Academic Editor

PLOS One

Additional Editor Comments (optional):

Reviewers' comments:

Reviewer's Responses to Questions

**Comments to the Author**

1. If the authors have adequately addressed your comments raised in a previous round of review and you feel that this manuscript is now acceptable for publication, you may indicate that here to bypass the “Comments to the Author” section, enter your conflict of interest statement in the “Confidential to Editor” section, and submit your "Accept" recommendation.

Reviewer #1: All comments have been addressed

**********

2. Is the manuscript technically sound, and do the data support the conclusions?

The manuscript must describe a technically sound piece of scientific research with data that supports the conclusions. Experiments must have been conducted rigorously, with appropriate controls, replication, and sample sizes. The conclusions must be drawn appropriately based on the data presented.

Reviewer #1: Yes

**********

3. Has the statistical analysis been performed appropriately and rigorously?

Reviewer #1: Yes

**********

4. Have the authors made all data underlying the findings in their manuscript fully available?

The PLOS Data policy requires authors to make all data underlying the findings described in their manuscript fully available without restriction, with rare exception (please refer to the Data Availability Statement in the manuscript PDF file). The data should be provided as part of the manuscript or its supporting information, or deposited to a public repository. For example, in addition to summary statistics, the data points behind means, medians and variance measures should be available. If there are restrictions on publicly sharing data—e.g. participant privacy or use of data from a third party—those must be specified.

Reviewer #1: Yes

**********

5. Is the manuscript presented in an intelligible fashion and written in standard English?

PLOS ONE does not copyedit accepted manuscripts, so the language in submitted articles must be clear, correct, and unambiguous. Any typographical or grammatical errors should be corrected at revision, so please note any specific errors here.

Reviewer #1: Yes

**********

6. Review Comments to the Author

Please use the space provided to explain your answers to the questions above. You may also include additional comments for the author, including concerns about dual publication, research ethics, or publication ethics. (Please upload your review as an attachment if it exceeds 20,000 characters)

Reviewer #1: Dear authors. I carefully went over the manuscript once more. I am pleased with the authors' efforts to revise the work. I believe that every query posed in the initial review has been addressed by the writers. The manuscript is now more comprehensive and easier to read. Its technical fit has improved in the interim. The manuscript as it stands, in my opinion, can advance knowledge. No further changes are required.

**********

7. PLOS authors have the option to publish the peer review history of their article (what does this mean?). If published, this will include your full peer review and any attached files.

If you choose “no”, your identity will remain anonymous but your review may still be made public.

**Do you want your identity to be public for this peer review?**  For information about this choice, including consent withdrawal, please see our Privacy Policy.

Reviewer #1: **Yes:** Gyanesh Kumar Tiwari

**********

# Editor report: Acceptance letter

**DOI:** 10.1371/journal.pone.0350918.r004

**Authors:** Giuseppe Marano

PONE-D-25-40092R1

PLOS One

Dear Dr. Lamb,

I'm pleased to inform you that your manuscript has been deemed suitable for publication in PLOS One. Congratulations! Your manuscript is now being handed over to our production team.

At this stage, our production department will prepare your paper for publication. This includes ensuring the following:

* All references, tables, and figures are properly cited

* All relevant supporting information is included in the manuscript submission,

* There are no issues that prevent the paper from being properly typeset

You will receive further instructions from the production team, including instructions on how to review your proof when it is ready. Please keep in mind that we are working through a large volume of accepted articles, so please give us a few days to review your paper and let you know the next and final steps.

Lastly, if your institution or institutions have a press office, please let them know about your upcoming paper now to help maximize its impact. If they'll be preparing press materials, please inform our press team within the next 48 hours. Your manuscript will remain under strict press embargo until 2 pm Eastern Time on the date of publication. For more information, please contact onepress@plos.org.

You will receive an invoice from PLOS for your publication fee after your manuscript has reached the completed accept phase. If you receive an email requesting payment before acceptance or for any other service, this may be a phishing scheme. Learn how to identify phishing emails and protect your accounts at https://explore.plos.org/phishing.

If we can help with anything else, please email us at customercare@plos.org.

Thank you for submitting your work to PLOS ONE and supporting open access.

Kind regards,

PLOS ONE Editorial Office Staff

on behalf of

Dr. Giuseppe Marano

Academic Editor

PLOS One
